# *Neospora caninum* infection specifically suppresses the expression of a host lncRNA *XR_001919077.1* to facilitate parasite propagation by modulating host cell mitochondrial function and autophagy

**DOI:** 10.1128/spectrum.01580-24

**Published:** 2024-12-23

**Authors:** Shan-Shan Zhao, De-Liang Tao, Jin-Ming Chen, Ming-Yi Zhang, Xin Yang, Jun-Ke Song, Qun Liu, Guang-Hui Zhao

**Affiliations:** 1 Department of Parasitology, College of Veterinary Medicine, Northwest A&F University, Yangling, Shaanxi, China; 2 National Animal Protozoa Laboratory, College of Veterinary Medicine, China Agricultural University, Beijing, China; Weill Cornell Medicine, New York, New York, USA

**Keywords:** *Neospora caninum*, *XR_001919077.1*, propagation, mitochondrial function, autophagy

## Abstract

*Neospora caninum* is one of the most common pathogens causing reproductive failure in ruminants (e.g., cattle and goats) worldwide. However, due to a poor understanding of the pathogenic mechanisms of *N. caninum* infection, no effective drugs and vaccines are currently available. Long non-coding RNAs (lncRNAs) have been reported to be important regulators involved in a great number of physiological and pathological processes. Our previous study found that *N. caninum* infection induced significantly aberrant expression of lncRNA profiles in caprine endometrial epithelial cells (EECs). In the present study, we found that *N. caninum* infection specifically suppressed the expression of a novel lncRNA, *XR_001919077.1*, and knockdown of *XR_001919077.1* with small interfering RNA significantly promoted the propagation of *N. caninum* in caprine EECs. Rapid amplification of cDNA ends analysis generated six splice variants of *XR_001919077.1*, with lengths ranging from 592 to 694 nt. Transfection of the full length of each variant markedly inhibited the propagation of *N. caninum* in caprine EECs. Further study suggested that *XR_001919077.1* acted as a sponge of Chi-miR-93-5p to promote the expression of *sirt1*, and the *XR_001919077.1*/Chi-miR-93-5p/*sirt1* axis significantly delayed the *in vitro* growth of *N. caninum* in caprine EECs by regulating host cell mitochondrial function and autophagy. Our findings provide a novel insight to understand the interactions between *N. caninum* and host cells.

**IMPORTANCE:**

The uterus is an indispensable reproductive organ for embryo implantation and fetal growth. The endometrium is more vulnerable to infection by pathogenic microorganisms resulting in an increased risk of miscarriage. *Neospora caninum* is one of the most common pathogens causing miscarriage in ruminants and is able to naturally inhabit the uterus, with *N. caninum* tissue cysts found in the endometrium. Recent advances in *N. caninum* research have revealed aberrant expression of long non-coding RNA (lncRNA) profiles in infected caprine endometrial epithelial cells. In the present study, *N. caninum*, but not *Toxoplasma gondii,* which has similar morphological and biological features to *N. caninum*, specifically suppresses the expression of a host lncRNA, *XR_ 001919077.1*, to impair host’s defense through the competitive endogenous RNA mechanism to modulate the host cell mitochondrial function and autophagy to facilitate parasite propagation. The findings suggest a novel immune evasion strategy of *N. caninum* to facilitate intracellular propagation and provide an alternative path to develop control strategies against neosporosis.

## INTRODUCTION

Neosporosis, caused by *Neospora caninum*, induces abortions and high neonatal mortality in cattle and small ruminants (e.g., goats and sheep) (1, 2). In the cattle industries of 10 countries, the estimated median losses due to *N. caninum* abortions exceed US$1,298.3 million (3). Unfortunately, no effective drugs are available to prevent and control neosporosis, and the only commercial vaccine (Neoguard, MSD Animal Health, Millsboro, DE, USA) against *N. caninum*-induced abortions has been withdrawn from the market due to low protective efficacy in infected cattle (4, 5). The main reason for the scarcity of available drugs and vaccines is due to a poor understanding of the pathogenic mechanisms of *N. caninum*.

Long non-coding RNAs (lncRNAs) are a class of non-translated RNA molecules that are usually transcribed by RNA polymerase II (Pol II)-dependent processes and have a length of more than 200 nucleotides (6). Recently, this type of non-coding RNA (ncRNA) has been demonstrated to be an important regulator involved in a great number of physiological and pathological processes (7–9). Previous studies showed that host cells could use lncRNAs to enhance defense against pathogenic infection, while the pathogens also could hijack lncRNAs to benefit their survival (10–15). For example, *Cryptosporidium parvum* infection induces the upregulated expression of a panel of lncRNAs (e.g., *NR_045064*, *U90926*, *XR_001779380*, *NR_033736,* and *Nostrill*), and these lncRNAs promote the transcription of defense genes through epigenetic modifications to enhance intestinal epithelial defense against *C. parvum* infection (10–14). The lncRNA *BTX* is significantly upregulated in peritoneal macrophages infected with vesicular stomatitis virus, and this lncRNA promotes viral replication by regulating the translocation of DHX9 and ILF3 from the nucleus to the cytoplasm (15). Our previous study showed that a total of 181 lncRNAs were differentially expressed in caprine endometrial epithelial cells (EECs) infected with *N. caninum* by RNA sequencing (RNA-seq) (16). However, the roles of these differentially expressed lncRNAs in the pathogenesis of neosporosis are still unknown. In the present study, we characterized one of the downregulated lncRNAs, namely *XR_001919077.1*, and investigated the effects and corresponding mechanisms of this lncRNA on the *in vitro* propagation of *N. caninum*.

## MATERIALS AND METHODS

### Parasites, cell lines, and *in vitro* infection model

*N. caninum* Nc-1 wild-type strain and *Toxoplasma gondii* RH strain were kindly gifted by Prof. Qun Liu from China Agricultural University (Beijing, China) and Assoc. Prof. Ningbo Xia from South China Agricultural University (Guangdong, China). *N. caninum* and *T. gondii* tachyzoites were serially passaged in African green monkey kidney epithelial cells (Vero cells). Caprine EECs were kindly provided by Prof. Yaping Jin from Northwest A&F University (Shaanxi, China) and were used for establishing an *in vitro* model of *N. caninum* infection at a multiplicity of infection (MOI) of 3:1 (parasite:cell) according to our previous study (17).

### RNA extraction and reverse transcriptase-quantitative polymerase chain reaction

Caprine EECs infected with *N. caninum* tachyzoites or *T. gondii* tachyzoites were collected, and the total RNA was isolated from collected cell samples using AG RNAex Pro Reagent (Accurate Biology, Hunan, China) following the manufacturer’s instructions. The concentration and quality of each RNA sample were assessed using a Nano-100 spectrophotometer (Allsheng Instruments Co., Hangzhou, China). RNA (1,000 ng) of each sample was reverse transcribed to cDNA using Hifair V Reverse Transcriptase (Yeasen Biotechnology Co., Ltd., Shanghai, China) for reverse transcriptase-quantitative polymerase chain reaction (RT-qPCR) analysis of mRNAs and lncRNAs, while 0.8 µg RNA of each sample was reverse transcribed using a Mir-X miRNA First-Strand Synthesis Kit (Takara Biomedical Technology, Dalian, China) for RT-qPCR analysis of microRNAs (miRNAs). The expression of mRNAs and lncRNAs was determined using 2× Universal SYBR Green Fast RT-qPCR Mix (ABclonal, Wuhan, China), with the *glyceraldehyde-3-phosphate dehydrogenase* (*gapdh*) gene as the internal reaction control. The expression of miRNAs was investigated using TB Green Fast qPCR Mix (Takara Biomedical Technology, Dalian, China), with the *u6* small nuclear RNA gene as the internal reaction control. Each reaction was performed in triplicate, and three replicates were done for each experiment. The relative abundance of each gene was calculated using the 2 ^−ΔΔCt^ method (18). The information on the primers used in this study is listed in Table S1.

### Rapid amplification of cDNA ends analysis and full-length amplification of *XR_001919077.1*

To obtain the 3′ and 5′ ends of unknown sequences of *XR_001919077.1*, 5′ and 3′ rapid amplification of cDNA ends (RACE) assays were performed using a SMARTer RACE 5′/3′ Kit (Clontech Laboratories, Inc., CA, USA) following the manufacturer’s protocol. The gene-specific primers used in RACE assays are listed in Table S1.

PCR primers (Table S1) were then designed based on 3′ and 5′ end sequences obtained from RACE assays to amplify the full length of *XR_001919077.1*. The PCR product was recovered, purified, ligated into a pMD19-T vector (Takara Biomedical Technology, Dalian, China), and transformed into *Escherichia coli* JM109 cells (Takara Biomedical Technology, Dalian, China). The positive colonies were sent to Tsingke Biotech (Beijing, China) for sequencing to determine the splice variants of *XR_001919077.1*.

### Construction of overexpression plasmids

The overexpression plasmid of each splice variant of *XR_001919077.1* was constructed into pcDNA 3.1 (+) vector (Invitrogen, Gaithersburg, MD, USA) using restriction endonucleases *Hin*d III and *Xho* I (Takara Biomedical Technology, Dalian, China). The overexpression plasmid of *sirt1* (pcDNA3.1 (+)-*sirt1*) was constructed into pcDNA 3.1 (+) vector by Tsingke Biotech (Beijing, China).

### Cell transfection

Caprine EECs were seeded into 12-well plates (Invitrogen, Waltham, MA, USA) for 24 h. Then, small interfering RNAs (siRNAs) (100 pmol/L, RiboBio Co., Ltd., Guangzhou, China) against *XR_001919077.1* (si-*XR_001919077.1*–1 and si-*XR_001919077.1*–2) or *sirt1* (si-*sirt1*), overexpression plasmid of each splice variant or *sirt1* (1.6 µg/well), or Chi-miR-93-5p mimics and inhibitor (60 pmol/L, GenePharma, Shanghai, China) were transfected into caprine EECs using Lipofectamine 2000 reagent (Invitrogen, Gaithersburg, MD, USA) according to the manufacturer’s instructions.

### Fluorescence *in situ* hybridization analysis

The subcellular location of *XR_001919077.1* was detected using Ribo Fluorescence *in Situ* Hybridization Kit (RiboBio Co., Ltd., Guangzhou, China) according to the manufacturer’s instructions. Briefly, caprine EECs were cultured onto glass coverslips in 24-well plates (Invitrogen, Waltham, MA, USA) for 24 h, and the cells were washed three times with PBS, fixed with 4% paraformaldehyde for 10 min at room temperature, permeabilized with PBS containing 0.5% Triton-X 100 at 4°C for 5 min, and blocked with prehybridization solution at 37°C for 30 min. The treated cells were incubated overnight at 4°C with rabbit anti-*N*. *caninum* MIC13 antibody (1:80) made in the parasitology laboratory of Northwest A&F University, Shaanxi, China. Secondary antibodies were FITC-conjugated donkey anti-rabbit IgG (1:200, Shanghai Sangon Biotech, Shanghai, China). The cells were then incubated with a hybridization solution containing a cy3-conjugated against *XR_001919077.1* probe (RiboBio Co., Ltd., Guangzhou, China) overnight at 37°C in the dark, with the *u6* probe (RiboBio Co., Ltd., Guangzhou, China) as a control. The nucleus was stained with 4′,6-diamidino-2-phenylindole (DAPI) for 10 min in the dark. The images were photographed under confocal immunofluorescence microscopy (Leica Microsystems, Wetzlar, Germany).

### Western blot analysis

Cell samples were lysed on ice in RIPA lysis buffer (Beijing Solarbio Science & Technology Co., Ltd., Beijing, China) supplemented with 1 mM phenylmethylsulfonyl fluoride (Beijing Solarbio Science & Technology Co., Ltd., Beijing, China), and the proteins were separated using sodium dodecyl sulfate-polyacrylamide gel and transferred to polyvinylidene difluoride membranes (Millipore, Billerica, USA). The membranes were blocked with 5% non-fat milk at room temperature for 2 h and then incubated with primary antibodies against SIRT1 (1:2,000, Abways, Shanghai, China), LC-3B (1:5,000, Abways, Shanghai, China), p62 (1:5,000, Abways, Shanghai, China), or β-actin (1:5,000, ABclonal, Wuhan, China) at 4°C overnight. Horseradish peroxidase-conjugated donkey anti-rabbit antibody (1:5,000, ABclonal, Wuhan, China) was used to incubate the membranes at room temperature for 1 h. Each reaction was performed in triplicate, and three replicates were done for each experiment. The protein blots were visualized using an enhanced chemiluminescence system, and gray values of the blots were calculated using ImageJ software (19).

### Dual-luciferase reporter assay

To determine the sponging relationship between *XR_001919077.1* and Chi-miR-93–5p, the sequence of *XR_001919077.1,* containing Chi-miR-93-5p binding site (WT) or mutated binding site (MUT), was cloned into the firefly luciferase pmirGLO reporter vector (Promega, Madison, WI, USA) to construct the recombinant plasmid pmirGLO-*XR_001919077.1-*WT or pmirGLO-*XR_001919077.1*-MUT. Caprine EECs were seeded into 96-well plates for 24 h, and the plasmid pmirGLO-*XR_001919077.1-*WT or pmirGLO-*XR_001919077.1*-MUT was transfected into caprine EECs in the presence of Chi-miR-93-5p mimics or control mimics using Lipofectamine 2000 reagent. Similarly, the 3′-untranslated region (UTR) sequences of *sirt1* containing Chi-miR-93-5p binding site (WT) or mutated binding site (MUT) were cloned into the firefly luciferase pmirGLO reporter vector to construct the recombinant plasmid pmirGLO-*sirt1*-WT or pmirGLO-*sirt1*-MUT and transfected into caprine EECs to verify the target relationship between Chi-miR-93-5p and *sirt1*. At 48 h post-transfection, the firefly luciferase activity was measured using a Luc-Pair Duo-Luciferase HS Assay Kit (GeneCopoeia Inc., MD, USA) on a multifunctional fluorimeter microplate reader (Tecan, Männedorf, Switzerland), with *Renilla* luciferase activity used as an internal reference. Each reaction was performed in triplicate, and three replicates were done for each experiment.

### Determination of reactive oxygen species, mitochondrial membrane potential, and adenosine triphosphate levels

The intracellular reactive oxygen species (ROS) production, mitochondrial membrane potential (MMP), and adenosine triphosphate (ATP) levels were, respectively, monitored using the 2′,7′-dichloro-fluorescin diacetate fluorescent probe (Beyotime Biotechnology, Shanghai, China), Mitochondrial Membrane Potential Assay Kit with JC-1 (Beyotime Biotechnology, Shanghai, China), and ATP Assay Kit (Beyotime Biotechnology, Shanghai, China) according to their manufacturer’s instructions. The fluorescence intensities of ROS and MMP were observed under an inverted fluorescence microscopy (Leica Microsystems, Wetzlar, Germany), and ATP contents were determined using a multifunctional fluorimeter microplate reader (Tecan, Männedorf, Switzerland) as previously described (20). Each reaction was performed in triplicate, and three replicates were done for each experiment.

### Determination of mitochondrial DNA copy numbers

The mitochondrial DNA (mtDNA) copy numbers were measured by quantifying the expression of the *nicotinamide adenine dinucleotide dehydrogenase subunit-1* (*nd1*) gene in genomic DNA (gDNA) (21). The gDNA samples were extracted from cell samples using a Blood/Cell/Tissue DNA Extraction Kit (Tiangen, Beijing, China) following the manufacturer’s protocol. A total of 200 ng gDNA was used as a template to perform RT-qPCR using 2× Universal SYBR Green Fast RT-qPCR Mix, with the *18s rRNA* gene as an internal reaction control. The primer sequences of the *nd1* gene and the *18s rRNA* gene are listed in Table S1. Each reaction was performed in triplicate, and three replicates were done for each experiment.

### Analysis of the propagation of *N. caninum* tachyzoites

Caprine EECs were infected with *N. caninum* tachyzoites at an MOI of 3:1 (parasite:cell) and cultured for 30 or 42 h post-infection (hpi). The number of *N. caninum* tachyzoites per parasitophorous vacuole was counted under inverted optical microscopy (Olympus Co., Tokyo, Japan).

The replication of *N. caninum* tachyzoites in the infected cells was also investigated based on the expression level of the *Nc5* gene of *N. caninum* using qPCR as described previously (22, 23). Briefly, the gDNA samples were extracted from 1 × 10^7^ *N. caninum* tachyzoites or infected cells using a Blood/Cell/Tissue DNA Extraction Kit (Tiangen, Beijing, China) following the manufacturer’s protocol. A total of 200 ng of gDNA samples from infected cells were used as templates to perform qPCR using 2× Universal SYBR Green Fast RT-qPCR Mix. The number of *N. caninum* tachyzoites in 200 ng gDNA samples was calculated based on a standard curve with gDNA samples from serial dilutions of *N. caninum* tachyzoites (5–5 × 10^5^ tachyzoites) included in each run. Each reaction was performed in triplicate, and three replicates were done for each experiment.

### Statistical analysis

Data obtained in each experiment were analyzed using the Student’s *t* test and one-way analysis of variance in GraphPad Prism 8.0.1 software (GraphPad Software Inc., San Diego, CA, USA). A *P* value < 0.05 was considered statistically significant.

## RESULTS

### Knockdown of *XR_001919077.1* significantly promotes the propagation of *N. caninum* in caprine EECs

**Fig 1:**
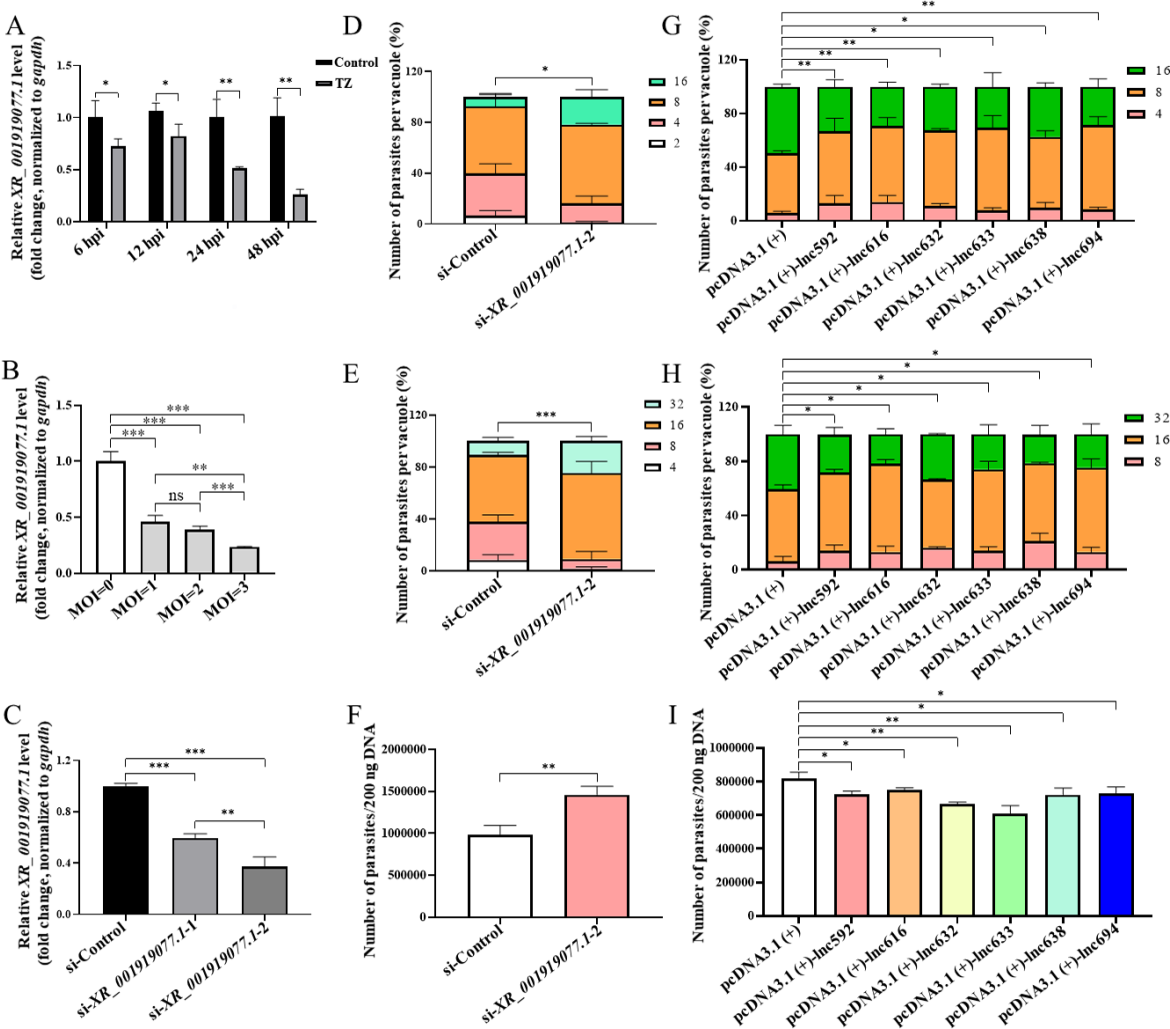
*XR_001919077.1* is negatively correlated with the propagation of *Neospora caninum* in caprine endometrial epithelial cells. (**A**) The expression of *XR_001919077.1* in caprine EECs during *N. caninum* infection for RT-qPCR. (**B**) The expression of *XR_001919077.1* in caprine EECs infected with different MOIs (parasite:cell = 0:1, 1:1, 2:1, 3:1) of *N. caninum* tachyzoites at 48 h post infection (hpi) for RT-qPCR. (**C**) Interference efficiencies of two small interfering RNAs (si-*XR_001919077.1*–1 and si-*XR_001919077.1*–2) against *XR_001919077.1* during *N. caninum* infection at 48 hpi for RT-qPCR. (**D and E**) Effect of knockdown of *XR_001919077.1* on the propagation of *N. caninum* tachyzoites in caprine EECs at 30 hpi (**D**) and 42 hpi (**E**). (**F**) Effect of knockdown of *XR_001919077.1* on the number of *N. caninum* in 200 ng DNA in infected caprine EECs at 48 hpi using qPCR. (**G and H**) Effect of overexpression of each *XR_001919077.1* variant on the propagation of *N. caninum* tachyzoites in caprine EECs at 30 hpi (**G**) and 42 hpi (**H**). (**I**) Effect of overexpression of each *XR_001919077.1* variant on the number of *N. caninum* in 200 ng DNA in infected caprine EECs at 48 hpi using qPCR. Data were obtained in triplicate and were analyzed using the Student’s *t* test. *P < 0.05, **P < 0.01, and ***P < 0.001. TZ represents the tachyzoite.

RT-qPCR analysis showed that the expression of *XR_001919077.1* was significantly downregulated from 6 to 48 hpi in caprine EECs infected with *N. caninum*, with the lowest expression level found at 48 hpi (Fig. 1A), and *N. caninum* infection further decreased the expression of *XR_001919077.1* in caprine EECs with the increase of MOIs at 48 hpi (Fig. 1B). However, *T. gondii* (MOI = 3:1), a pathogen with similar morphological and biological features to *N. caninum*, did not significantly alter the expression of *XR_001919077.1* in caprine EECs at 48 hpi (Fig. S1A), and the expression of *XR_001919077.1* was not affected in caprine EECs stimulated with lipopolysaccharide (200 nM; Abmole, Shanghai, China) (Fig. S1B) for 48 h. These findings suggest that *N. caninum* may specifically suppress the expression of *XR_001919077.1* in caprine EECs.

To determine the role of *XR_001919077*.1 during *N. caninum* infection, two siRNAs against *XR_001919077.1* (si-*XR_001919077.1*-1 and si-*XR_001919077.1*-2) were designed, and the expression of *XR_001919077*.1 was significantly decreased by both si-*XR_001919077.1*-1 and si-*XR_001919077.1*-2, but the interference effect of si-*XR_001919077.1*-2 (∼70%) was more profound (*P* < 0.01) than si-*XR_001919077.1*-1 (∼30%) (Fig. 1C). Therefore, si-*XR_001919077.1*-2 was used for further study. Interestingly, transfection with si-*XR_001919077.1*-2 significantly increased the number of *N. caninum* tachyzoites per vacuole in caprine EECs at 30 hpi (Fig. 1D) and 42 hpi (Fig. 1E). qPCR of the *Nc5* gene showed that the number of *N. caninum* was also dramatically increased in infected caprine EECs treated with si-*XR_001919077.1*-2 at 48 hpi (Fig. 1F). These findings suggest that the expression of *XR_001919077.1* is negatively correlated with the growth and propagation of *N. caninum* tachyzoites *in vitro*.

### *XR_001919077.1* expresses six splice variants

The 5′ and 3′ RACE assays of *XR_001919077.1* obtained two sequences and one sequence with a Poly-A tail, respectively (Fig. S2). To obtain the full length of *XR_001919077.1*, two pairs of primers (Table S1) were designed based on the sequences from the 5′ and 3′ RACE assays and used for PCR amplification and subsequent sequencing. Sequence analysis showed that six splice variants were generated by *XR_001919077.1*, named lnc592, lnc616, lnc632, lnc633, lnc638, and lnc694 according to their lengths (Fig. S3). The coding potential of each variant was investigated using CPC 2.0, but none of them had coding potential (data not shown). Notably, si-*XR_001919077.1*-2 could partially inhibit the expression of each *XR_001919077.1* variant in caprine EECs infected with *N. caninum* (Fig. S4A).

### Overexpression of *XR_001919077.1* inhibits the propagation of *N. caninum in vitro*

To further examine the role of *XR_001919077.1* on the propagation of *N. caninum*, the recombinant plasmids of six *XR_001919077.1* variants, namely pcDNA3.1 (+)-lnc592, pcDNA3.1 (+)-lnc616, pcDNA3.1 (+)-lnc632, pcDNA3.1 (+)-lnc633, pcDNA3.1 (+)-lnc638, and pcDNA3.1 (+)-lnc694, were transfected into caprine EECs at 24 h before infection of *N. caninum*, respectively. RT-qPCR showed that the expression of each *XR_001919077.1* variant was significantly upregulated after transfection (Fig. S4B). Notably, overexpression of each of the six variants significantly decreased the number of *N. caninum* tachyzoites per vacuole at 30 hpi (Fig. 1G) and 42 hpi (Fig. 1H). qPCR of the *Nc5* gene showed that the number of *N. caninum* was also dramatically decreased in infected caprine EECs transfected with each variant of *XR_001919077.1* at 48 hpi (Fig. 1I). These results suggest that *XR_001919077.1* significantly inhibits intracellular propagation of *N. caninum* tachyzoites in caprine EECs.

### *XR_001919077.1* promotes the expression of *sirt1* by sponging Chi-miR-93-5p in caprine EECs infected with *N. caninum*

**Fig 2:**
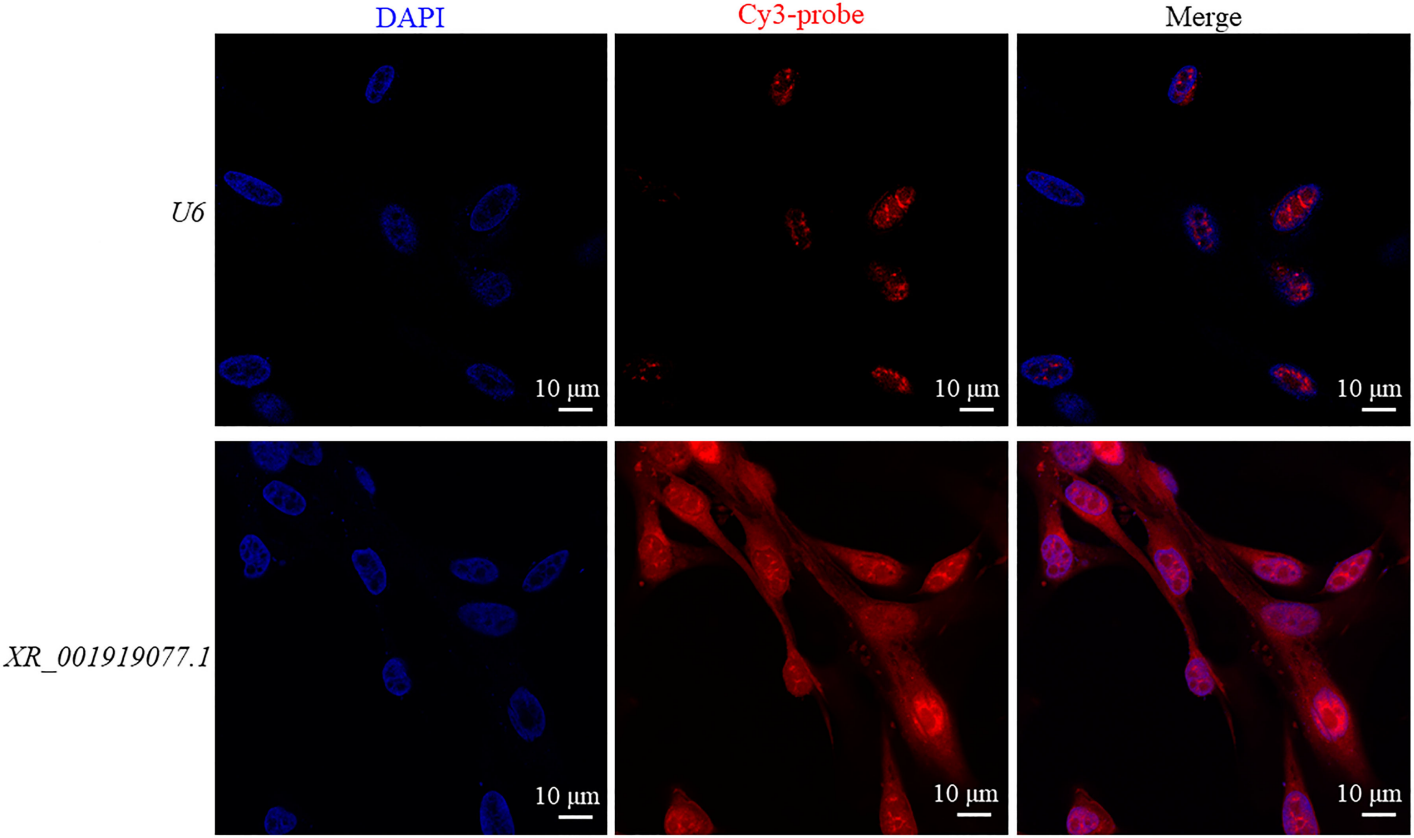
*XR_001919077.1* localizes in both cytoplasm and nucleus in caprine endometrial epithelial cells. The nucleus was stained with DAPI. Scale bar: 10 µm.

To investigate the mechanisms of *XR_001919077.1* that affect the intracellular propagation of *N. caninum* tachyzoites *in vitro*, the location of *XR_001919077.1* in caprine EECs was investigated using a cy3-conjugated probe against *XR_001919077.1*, with *u6* localized in the nucleus as a control. Fluorescence *in situ* hybridization (FISH) analysis showed that *XR_001919077.1* was found in both cytoplasm and nucleus in caprine EECs with or without infection of *N. caninum* at 48 hpi (Fig. 2; Fig. S5).

**Fig 3:**
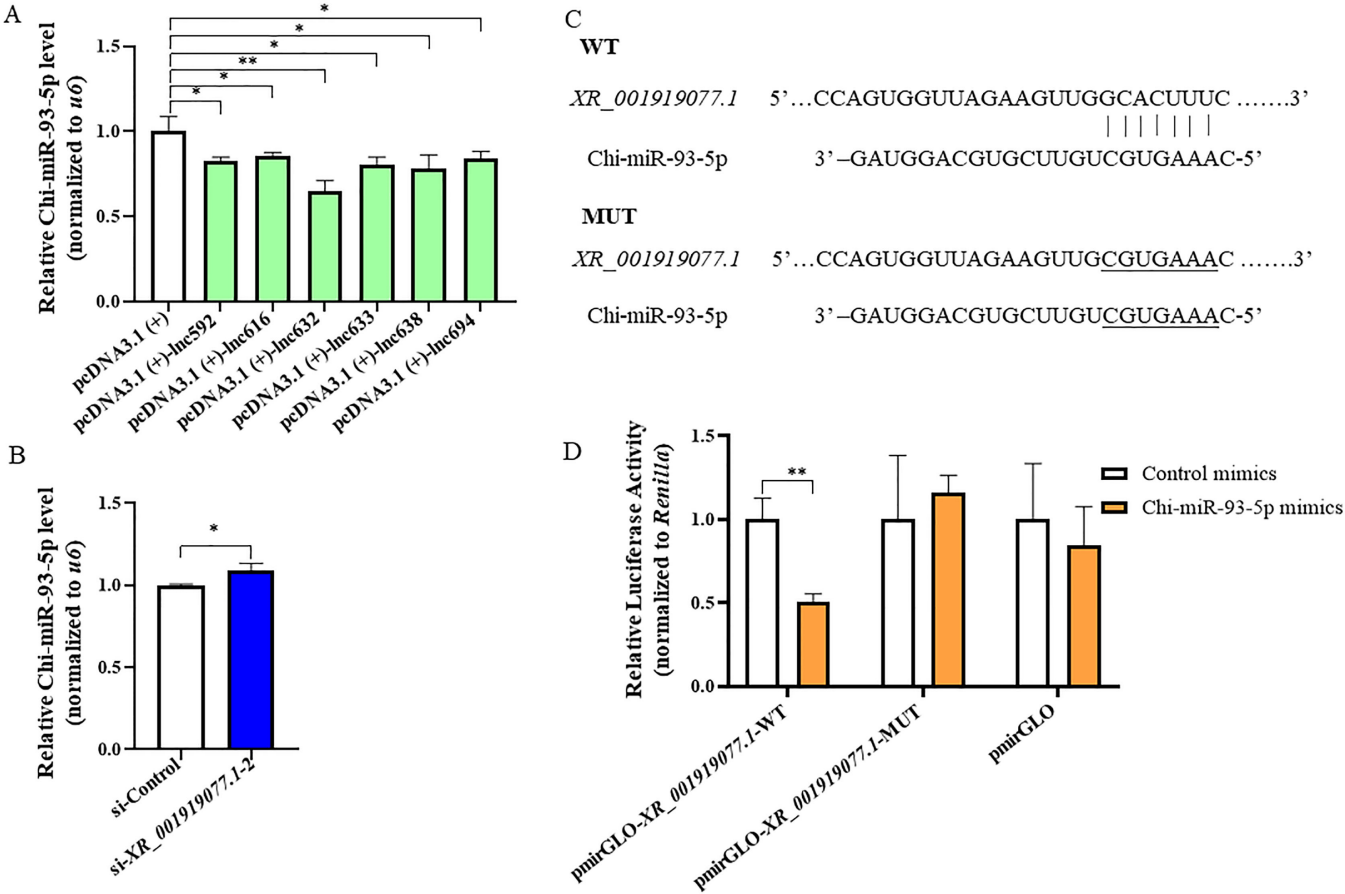
*XR_001919077.1* functions as a sponge for Chi-miR-93-5p in caprine endometrial epithelial cells infected with *N. caninum*. (**A**) The expression of Chi-miR-93-5p in caprine EECs infected with *N. caninum* by overexpression of each *XR_001919077.1* variant at 48 h post-infection (hpi). (**B**) The expression of Chi-miR-93-5p in caprine EECs infected with *N. caninum* by interference with si-*XR_001919077.1*-2 at 48 hpi. (**C**) Potential binding sites between *XR_001919077.1* and Chi-miR-93-5p predicted by miRanda database. (**D**) Sponging relationship between *XR_001919077.1* and Chi-miR-93-5p identified using the dual-luciferase reporter assay. Data were obtained in triplicate and were analyzed using the Student’s *t* test. *P < 0.05 and **P < 0.01.

The most common function of lncRNAs is to act as competitive endogenous RNAs (ceRNAs) to regulate the expression of targeted genes through a competitive combination of miRNAs (24, 25). Bioinformatics analysis using the miRanda database identified 18 potential sponging miRNAs for *XR_001919077.1*. RT-qPCR showed that six (Chi-miR-30e-5p, Chi-miR-495-3p, Chi-miR-93-5p, Chi-miR-211, Chi-miR-134, and Chi-miR-128–5p) of them were significantly upregulated in caprine EECs infected with *N. caninum* at 48 hpi (Fig. S6A). However, the expression of only one miRNA, named Chi-miR-93-5p, was significantly inhibited by overexpression of each *XR_001919077.1* variant (Fig. 3A; Fig. S6B), while knockdown of *XR_001919077.1* using si-*XR_001919077.1*-2 promoted the expression of Chi-miR-93-5p during *N. caninum* infection (Fig. 3B). The dual-luciferase reporter assay, based on predicting potential binding sites between *XR_001919077.1* and Chi-miR-93-5p (Fig. 3C), showed that Chi-miR-93-5p mimics markedly reduced the luciferase activity of the pmirGLO-*XR_001919077.1-*WT vector but did not affect that of the pmirGLO-*XR_001919077.1*-MUT vector in caprine EECs (Fig. 3D). These results suggest the sponging relationship between *XR_001919077.1* and Chi-miR-93-5p in caprine EECs during *N. caninum* infection.

**Fig 4:**
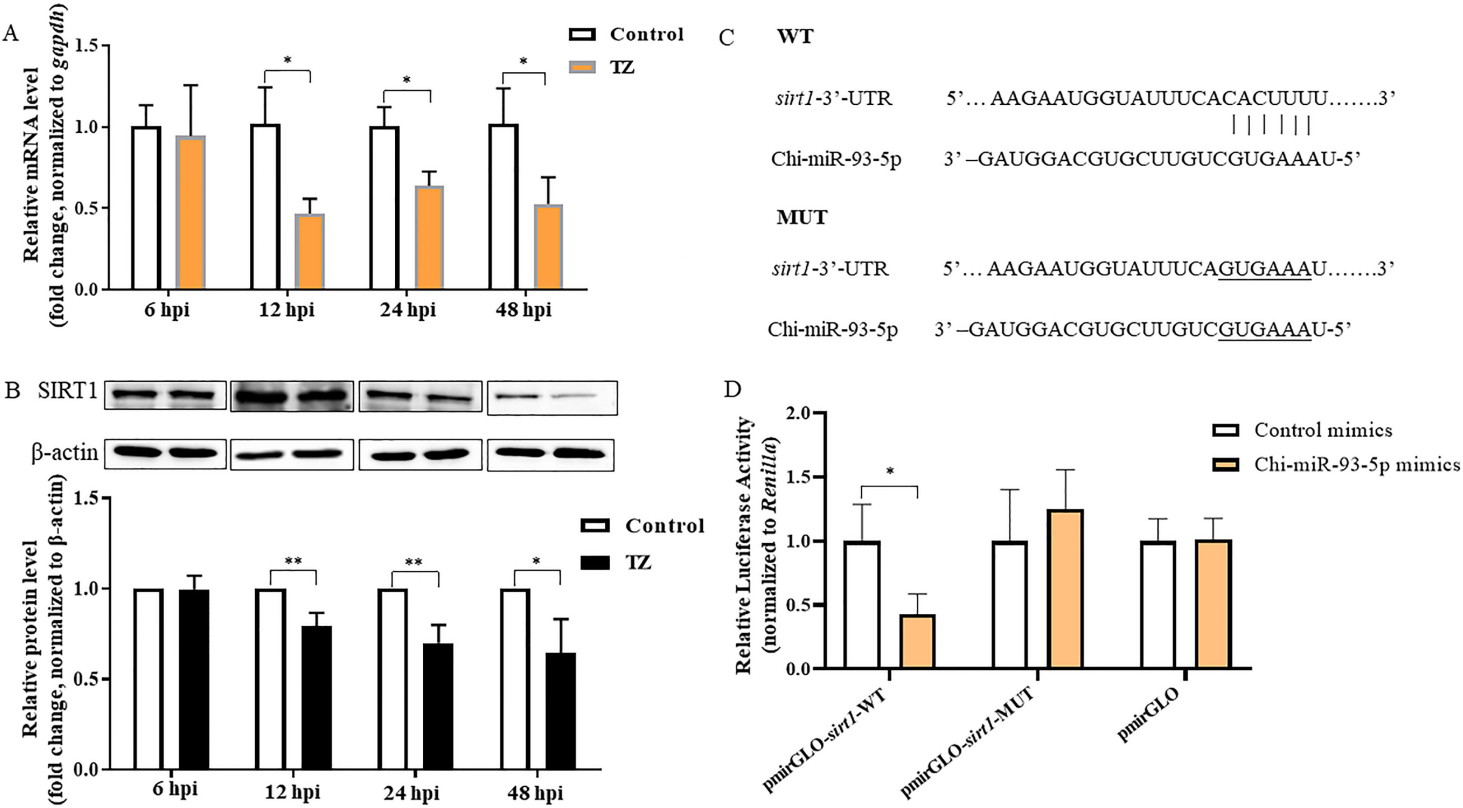
Chi-miR-93-5p targets *sirt1* in caprine endometrial epithelial cells infected with *N. caninum*. (**A**) mRNA level of *sirt1* analyzed in caprine EECs infected with *N. caninum* tachyzoites from 6 to 48 h post infection (hpi) by RT-qPCR. (**B**) Protein level of *sirt1* analyzed in caprine EECs infected with *N. caninum* tachyzoites from 6 to 48 hpi by western blotting. Relative protein levels of SIRT1 compared to β-actin were determined by densitometry. (**C**) Potential binding sites between Chi-miR-93-5p and 3′-UTR of *sirt1*. (**D**) Target relationship between *sirt1* and Chi-miR-93-5p identified using the dual-luciferase reporter assay. Data were obtained in triplicate and were analyzed using the Student’s *t* test. *P < 0.05 and **P < 0.01. TZ represents the tachyzoite.

**Fig 5:**
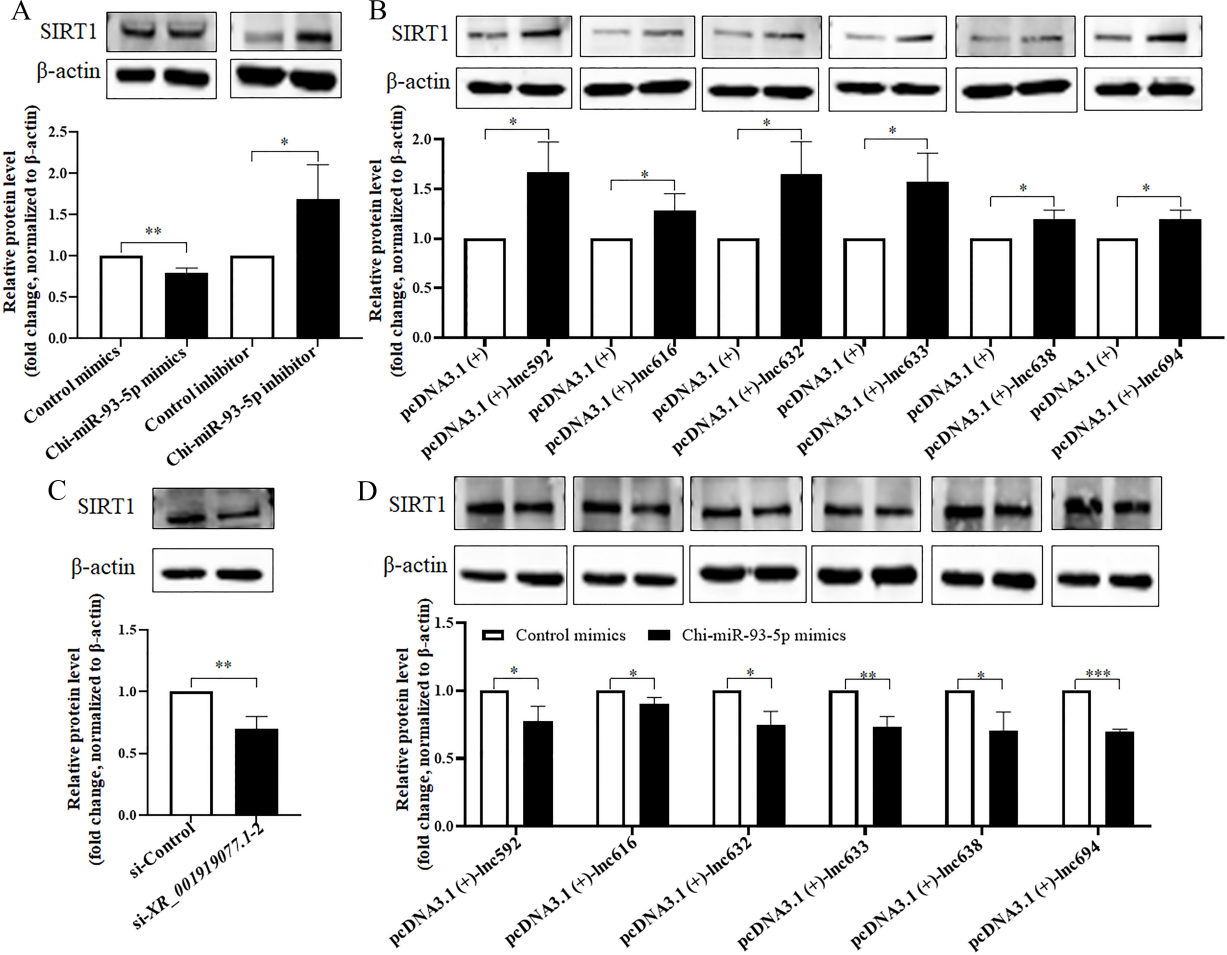
*XR_001919077.1* promotes the expression of *sirt1* by sponging Chi-miR-93-5p in caprine endometrial epithelial cells following *N. caninum* infection. (**A**) The expression of the SIRT1 protein analyzed in caprine EECs transfected with Chi-miR-93-5p mimics or inhibitor during *N. caninum* infection at 48 h post-infection (hpi) using western blotting. (**B**) The expression of the SIRT1 protein analyzed in caprine EECs transfected with recombinant plasmids of six *XR_001919077.1* variants during *N. caninum* infection at 48 hpi using western blotting. (**C**) The expression of the SIRT1 protein analyzed in caprine EECs transfected with si-*XR_001919077.1*–2 during *N. caninum* infection at 48 hpi using western blotting. (**D**) The expression of the SIRT1 protein analyzed in caprine EECs co-transfected with recombinant plasmid of each of six *XR_001919077.1* variants and Chi-miR-93-5p mimics during *N. caninum* infection at 48 hpi using western blotting. Relative protein level of SIRT1 compared to β-actin was determined by densitometry. Data were obtained in triplicate and were analyzed using the Student’s *t* test. *P < 0.05, **P < 0.01, and ***P < 0.001.

SIRT1, a conserved protein NAD (+)-dependent deacetylases, is a key modulator involved in metabolic and various physiological processes, including senescence, inflammatory response, and mitochondrial biogenesis, and has been identified as a target for Chi-miR-93-5p in rats with type 2 diabetic retinopathy, old-age rats, and human dermal fibroblasts (26–28). Coincidentally, our previous study showed that *N. caninum* infection suppressed the expression of SIRT1 in caprine EECs (20). In the present study, the mRNA and protein levels of *sirt1* were also significantly downregulated from 12 to 48 hpi during *N. caninum* infection (Fig. 4A and B). To verify the targeting relationship between Chi-miR-93-5p and *sirt1* in caprine EECs, the dual-luciferase reporter assay was performed based on predicting potential binding sites between Chi-miR-93-5p and *sirt1* 3′-untranslated region (Fig. 4C). Chi-miR-93-5p mimics significantly inhibited the luciferase activity of the pmirGLO-*sirt1*-WT but did not affect that of the pmirGLO-*sirt1*-MUT in caprine EECs (Fig. 4D). To further determine the effects of Chi-miR-93-5p on the expression of *sirt1* during *N. caninum* infection, Chi-miR-93-5p mimics or Chi-miR-93-5p inhibitor was transfected into caprine EECs. In caprine EECs infected with *N. caninum*, the expression of Chi-miR-93-5p was significantly increased by Chi-miR-93-5p mimics but was significantly decreased by Chi-miR-93-5p inhibitor (Fig. S7). Meantime, Chi-miR-93-5p mimics significantly decreased both the mRNA (Fig. S8A) and protein (Fig. 5A) levels of *sirt1*, while the reverse effects were detected for Chi-miR-93-5p inhibitor (Fig. 5A; Fig. S8A). These data suggest that Chi-miR-93-5p could directly target *sirt1* in caprine EECs during *N. caninum* infection.

Interestingly, overexpression of each *XR_001919077.1* variant significantly increased the mRNA (Fig. S8B) and protein levels (Fig. 5B) of *sirt1*, but knockdown of *XR_001919077.1* using si-*XR_001919077.1*-2 significantly decreased the mRNA (Fig. S8C) and protein levels (Fig. 5C) of *sirt1*. Furthermore, compared with control mimics, co-transfection of Chi-miR-93-5p mimics with each of six *XR_001919077.1* variants significantly decreased the protein level of SIRT1 (Fig. 5D). These results suggest that *XR_001919077.1* promotes the expression of *sirt1* by sponging Chi-miR-93-5p in caprine EECs during *N. caninum* infection.

### *XR_001919077.1* delays *in vitro* propagation of *N. caninum* tachyzoites by regulating the Chi-miR-93-5p/*sirt1* axis

**Fig 6:**
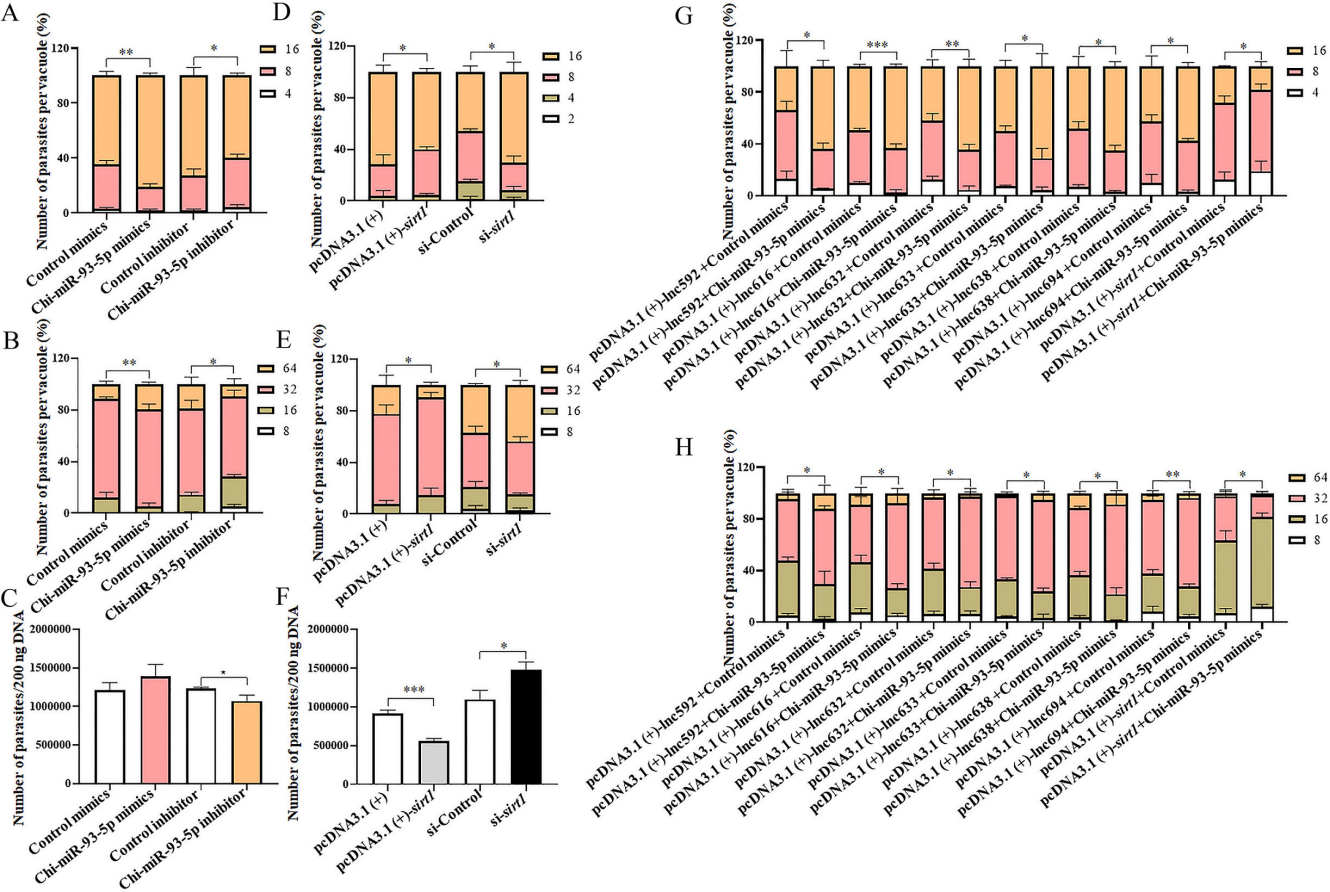
The *XR_001919077.1*/Chi-miR-93-5p/*sirt1* axis delays the propagation of *N. caninum* tachyzoites in caprine endometrial epithelial cells. (**A and B**) Effect of Chi-miR-93-5p mimics or inhibitor on the propagation of *N. caninum* tachyzoites in caprine EECs at 30 h post infection (hpi) (**A**) and 42 hpi (**B**). (**C**) The number of *N. caninum* in 200 ng DNA in infected caprine EECs transfected with Chi-miR-93-5p mimics or inhibitor at 48 hpi using qPCR. (**D and E**) Effect of pcDNA3.1 (+)-*sirt1* or si-*sirt1* on the propagation of *N. caninum* tachyzoites in caprine EECs at 30 hpi (**D**) and 42 hpi (**E**). (**F**) The number of *N. caninum* in 200 ng DNA in infected caprine EECs transfected with pcDNA3.1 (+)-*sirt1* or si-*sirt1* at 48 hpi using qPCR. (**G and H**) Effect of co-transfection of Chi-miR-93-5p mimics and each of the six *XR_001919077.1* variants, or pcDNA3.1(+)-*sirt1* on the propagation of *N. caninum* tachyzoites in caprine EECs at 30 hpi (**G**) and 42 hpi (**H**). Data were obtained in triplicate and were analyzed using the Student’s *t* test. *P < 0.05, **P < 0.01, and ***P < 0.001.

In caprine EECs, Chi-miR-93-5p mimics significantly increased the number of *N. caninum* tachyzoites per vacuole at 30 hpi (Fig. 6A) and 42 hpi (Fig. 6B), while the opposite effect on the infection burden of this parasite was detected for Chi-miR-93-5p inhibitor at 30 hpi (Fig. 6A) and 42 hpi (Fig. 6B). qPCR of the *Nc5* gene showed that although the number of *N. caninum* was not significantly increased in infected caprine EECs transfected with Chi-miR-93-5p mimics at 48 hpi, the number of *N. caninum* was significantly decreased for transfection of Chi-miR-93-5p inhibitor (Fig. 6C). Our previous study found that EX527, an inhibitor of SIRT1, promoted the propagation of *N. caninum* tachyzoites in caprine EECs, while resveratrol (an activator of SIRT1) inhibited the *in vitro* propagation of *N. caninum* tachyzoites (20). To exclude extensive pharmacological effects, an overexpression plasmid, pcDNA3.1 (+)-*sirt1,* was constructed, and a siRNA, si-*sirt1*, was synthesized in the present study. In caprine EECs infected with *N. caninum*, the mRNA and protein levels of *sirt1* were significantly upregulated by transfection with pcDNA3.1 (+)-*sirt1* (Fig. S9) but were significantly downregulated by transfection with si-*sirt1* (Fig. S9). Meantime, overexpression of *sirt1* significantly decreased the number of *N. caninum* tachyzoites per vacuole at 30 hpi (Fig. 6D) and 42 hpi (Fig. 6E), while the opposite effect on the infection burden of this parasite was detected for si-*sirt1* at 30 hpi (Fig. 6D) and 42 hpi (Fig. 6E). qPCR of the *Nc5* gene showed that the number of *N. caninum* was dramatically decreased in infected caprine EECs treated with overexpression plasmids of pcDNA3.1 (+)-*sirt1* at 48 hpi, while the opposite effect was detected for si-*sirt1* (Fig. 6F). Furthermore, compared with the control mimics, co-transfection of Chi-miR-93-5p mimics and pcDNA3.1(+)-*sirt1* inhibited the propagation of *N. caninum* tachyzoites in caprine EECs, but co-transfection of Chi-miR-93-5p mimics with each of the six *XR_001919077.1* variants significantly promoted the propagation of this parasite in caprine EECs (Fig. 6G and H). These results suggest that the expression of *XR_001919077.1* is negatively correlated with the propagation of *N. caninum* by promoting the expression of *sirt1* by sponging Chi-miR-93-5p in caprine EECs.

### *XR_001919077.1*/Chi-miR-93-5p/*sirt1* axis regulates mitochondrial function in caprine EECs during *N. caninum* infection

**Fig 7:**
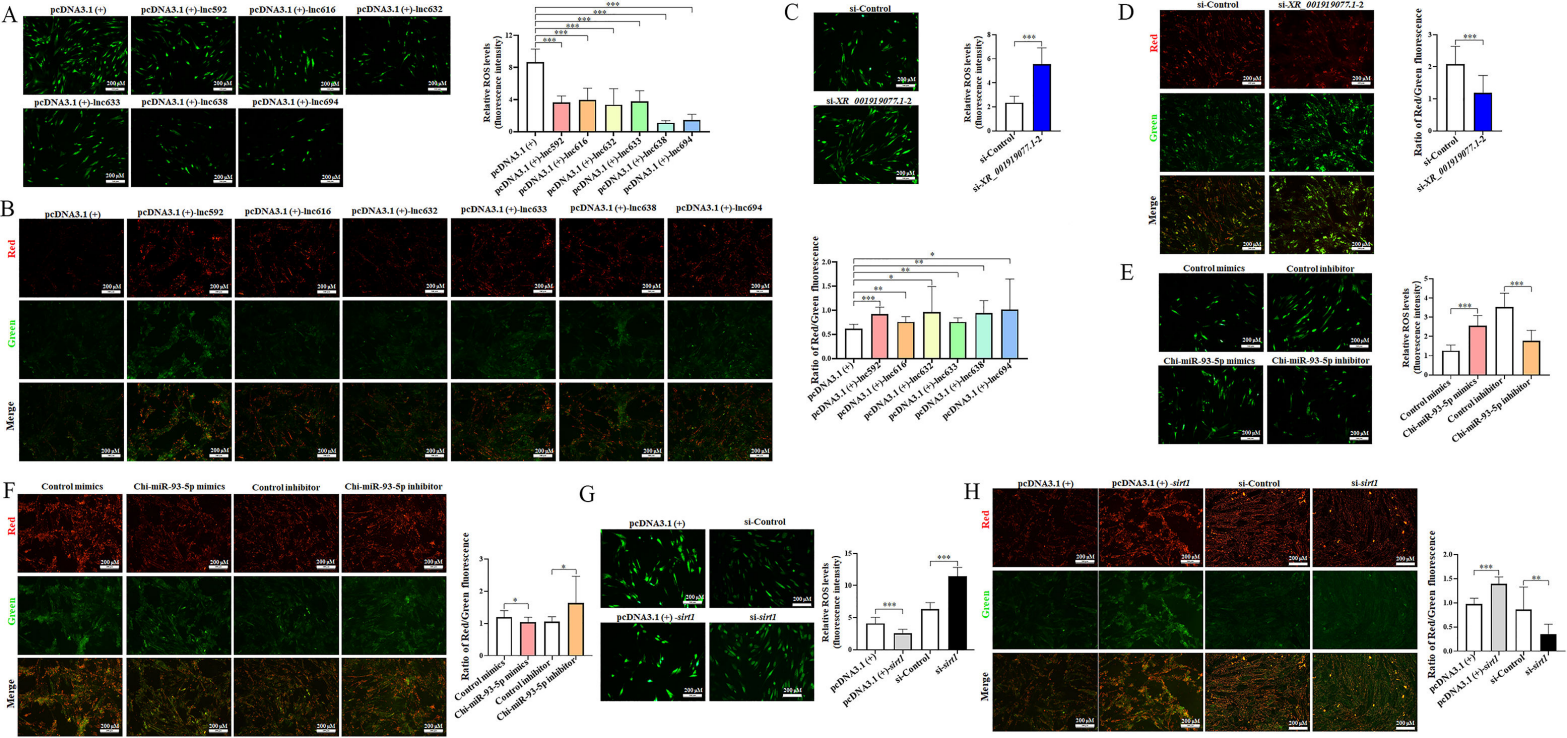
The *XR_001919077.1*/Chi-miR-93-5p/*sirt1* axis affects the ROS levels and MMP levels in caprine EECs following *N. caninum* infection. (**A and B**) ROS levels (**A**) and MMP levels (**B**) determined in caprine EECs transfected with recombinant plasmids of six *XR_001919077.1* variants during *N. caninum* infection at 48 h post-infection (hpi). (**C and D**) ROS levels (**C**) and MMP levels (**D**) determined in caprine EECs transfected with si-*XR_001919077.1*-2 during *N. caninum* infection at 48 hpi. (**E and F**) ROS levels (**E**) and MMP levels (**F**) determined in caprine EECs transfected with Chi-miR-93-5p mimics or inhibitor during *N. caninum* infection at 48 hpi. (**G and H**) ROS levels (**G**) and MMP levels (**H**) determined in caprine EECs transfected with pcDNA3.1 (+)-*sirt1* or si-*sirt1* during *N. caninum* infection at 48 hpi. Data were obtained in triplicate and were analyzed using the Student’s *t* test. Scale bar: 200 µm. *P < 0.05, **P < 0.01, and ***P < 0.001.

**Fig 8:**
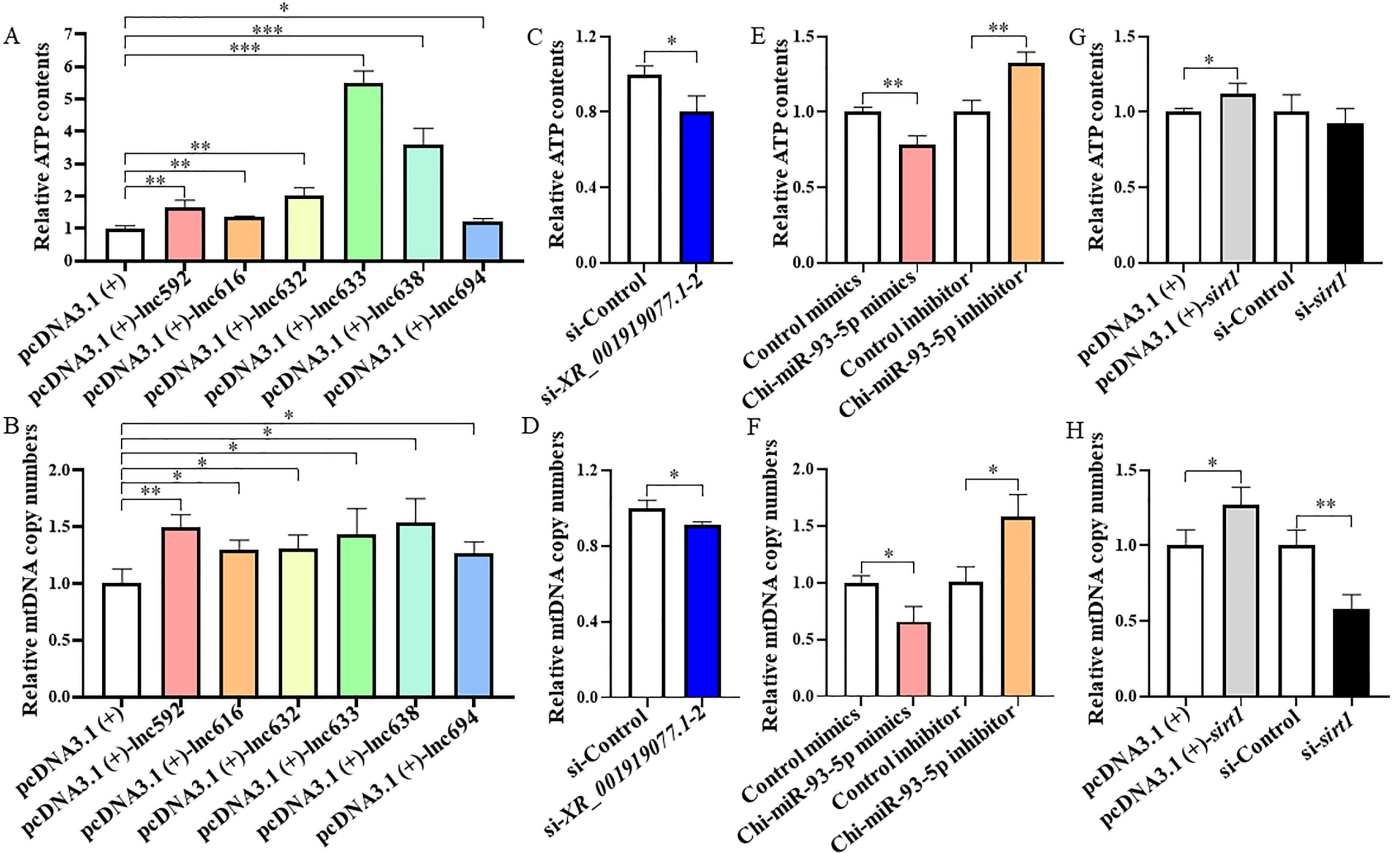
The *XR_001919077.1*/Chi-miR-93-5p/*sirt1* axis affects the adenosine triphosphate (ATP) contents and mtDNA copy numbers in caprine endometrial epithelial cells following *N. caninum* infection. (**A and B**) ATP contents (**A**) and mtDNA copy numbers (**B**) determined in caprine EECs transfected with recombinant plasmids of six *XR_001919077.1* variants during *N. caninum* infection at 48 h post-infection (hpi). (**C and D**) ATP contents (**C**) and mtDNA copy numbers (**D**) determined in caprine EECs transfected with si-*XR_001919077.1*-2 during *N. caninum* infection at 48 hpi. (**E and F**) ATP contents (**E**) and mtDNA copy numbers (**F**) determined in caprine EECs transfected with Chi-miR-93-5p mimics or inhibitor during *N. caninum* infection at 48 hpi. (**G and H**) ATP contents (**G**) and mtDNA copy numbers (**H**) determined in caprine EECs transfected with pcDNA3.1 (+)-*sirt1* or si-*sirt1* during *N. caninum* infection at 48 hpi. Data were obtained in triplicate and were analyzed using the Student’s *t* test. Scale bar: 200 µm. *P < 0.05, **P < 0.01, and ***P < 0.001.

Our previous study found that *N. caninum* infection downregulated the expression of SIRT1 to facilitate the propagation by damaging the mitochondrial function (20). In the present study, we asked whether the *XR_001919077.1*/Chi-miR-93–5p*/sirt1* axis delayed the *in vitro* propagation of *N. caninum* by regulating mitochondrial function. To address this issue, caprine EECs were respectively transfected with each of the six *XR_001919077.1* variants, si-*XR_001919077.1*-2, Chi-miR-93-5p mimics or inhibitor, pcDNA3.1 (+)-*sirt1,* or si-*sirt1*, and then infected with *N. caninum* at 24 h post-transfection. Overexpression of each variant of *XR_001919077.1* significantly decreased ROS production (Fig. 7A) but increased MMP levels (Fig. 7B), ATP contents (Fig. 8A), and mtDNA copy numbers (Fig. 8B) in infected caprine EECs, while opposite effects were detected for si-*XR_001919077.1*-2 (Fig. 7C, D, 8C, and D). Transfection of Chi-miR-93-5p mimics induced the accumulation of intracellular ROS (Fig. 7E) and a marked reduction of MMP levels (Fig. 7F), ATP contents (Fig. 8E), and mtDNA copy numbers (Fig. 8F) in infected caprine EECs. However, contrary results were detected for the transfection of Chi-miR-93–5p inhibitor (Fig. 7E, F, 8E, and F). The effects for intracellular ROS, MMP levels, ATP contents, and mtDNA copy numbers induced by overexpression of *sirt1* were opposite to transfection of Chi-miR-93-5p mimics but were consistent with transfection of each of the six *XR_001919077.1* variants (Fig. 7G, H, 8G, and H), while the opposite effects were observed for si-*sirt1* (Fig. 7G, H, 8G, and H). These results suggest that *N. caninum* infection induces mitochondrial dysfunction to facilitate intracellular propagation through the *XR_001919077.1*/Chi-miR-93–5p/*sirt1* axis.

### *XR_001919077.1*/Chi-miR-93-5p/*sirt1* axis regulates cell autophagy in caprine EECs during *N. caninum* infection

**Fig 9:**
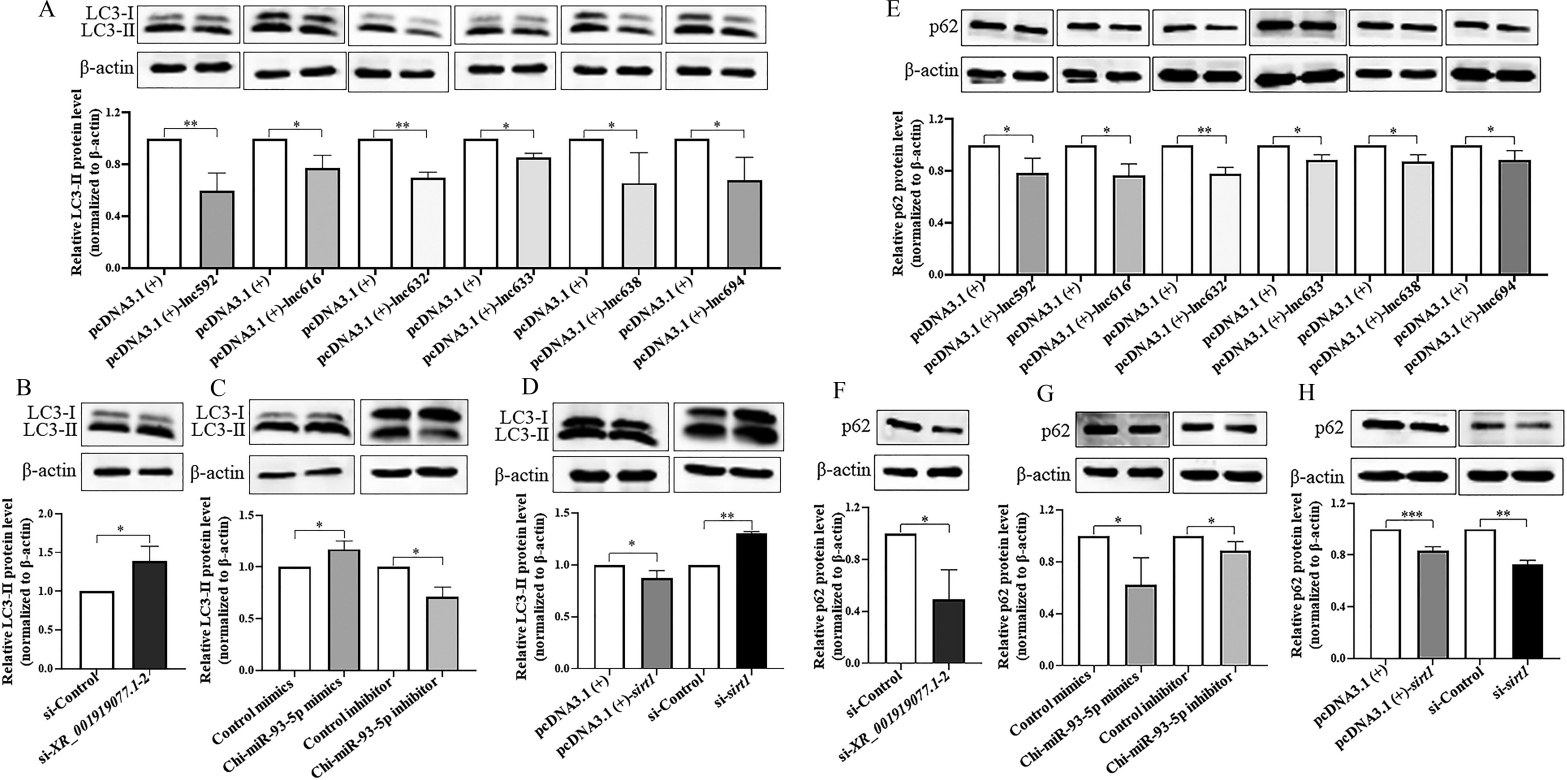
The *XR_001919077.1*/Chi-miR-93–5p/*sirt1* axis affects the expression of the LC3B protein and p62 protein in caprine endometrial epithelial cells following *N. caninum* infection. (**A–D**) The protein levels of LC3B determined in caprine EECs transfected with recombinant plasmids of the six *XR_001919077.1* variants (**A**), si-*XR_001919077.1*-2 (**B**), Chi-miR-93-5p mimics or inhibitor (**C**), pcDNA3.1 (+)-*sirt1,* or si-*sirt1* (**D**) during *N. caninum* infection at 48 h post-infection (hpi) by western blotting. (**E–H**) The protein levels of p62 determined in caprine EECs transfected with recombinant plasmids of the six *XR_001919077.1* variants (**E**), si-*XR_001919077.1*–2 (**F**), Chi-miR-93-5p mimics or inhibitor (**G**), pcDNA3.1 (+)-*sirt1,* or si-*sirt1* (**H**) during *N. caninum* infection at 48 hpi by western blotting. The relative protein levels of LC3-II (**A–D**) or p62 (**E–H**) compared to β-actin were determined by densitometry. Data were obtained in triplicate and were analyzed using the Student’s *t* test. *P < 0.05, **P < 0.01, and ***P < 0.001.

Our previous studies showed that *N. caninum* infection inhibited the expression of *sirt1* to promote autophagy in caprine EECs (20). We then asked whether cell autophagy induced by *N. caninum* infection was regulated by the *XR_001919077.1*/Chi-miR-93-5p/*sirt1* axis. In the present study, caprine EECs were transfected with each of the six *XR_001919077.1* variants, si-*XR_001919077.1*-2, Chi-miR-93-5p mimics or inhibitor, pcDNA3.1 (+)-*sirt1,* or si-*sirt1*, and then infected with *N. caninum* at 24 h post-transfection. The protein levels of LC3-II (a marker of autophagosome formation) and p62 (an autophagy receptor protein involved in the degradation of ubiquitinated cargo and a common marker for monitoring the occurrence of autophagy flow) were examined at 48 hpi. Transfection with each *XR_001919077.1* variant significantly decreased the expression of LC3-II protein (Fig. 9A), while the opposite effect was detected for transfection with si-*XR_001919077.1*-2 (Fig. 9B). Increased expression of LC3-II protein was found following transfection with Chi-miR-93-5p mimics, but the contrary result was detected following transfection with Chi-miR-93-5p inhibitor (Fig. 9C). Additionally, transfection with pcDNA3.1 (+)-*sirt1* significantly decreased the protein level of LC3-II, but the contrary result was detected for transfection with si-*sirt1* (Fig. 9D). However, the protein levels of p62 decreased upon transfection of each *XR_001919077.1* variant (Fig. 9E), si-*XR_001919077.1*–2 (Fig. 9F), Chi-miR-93–5p mimics and inhibitor (Fig. 9G), pcDNA3.1 (+)-*sirt1,* and si-*sirt1* (Fig. 9H) in caprine EECs during *N. caninum* infection. These results suggest that the *XR_001919077.1*/Chi-miR-93–5p/*sirt1* axis inhibits autophagy in caprine EECs induced by *N. caninum* infection.

## DISCUSSION

Reproductive failure in pregnant animals is the main consequence induced by *N. caninum* infection (1). Therefore, uncovering the intracellular survival and pathogenicity of *N. caninum* in reproductive organs is essential to exploring novel effective targets for controlling neosporosis. The uterus, an indispensable reproductive organ that forms the mother-fetal interface, embryo implantation, and maintenance of gestation, has been reported naturally inhabited by *N. caninum* (29–31). In the present study, our data indicate that a host lncRNA, namely *XR_001919077.1*, is specifically significantly downregulated during *N. caninum* infection, and the *XR_001919077.1*/Chi-miR-93-5p/*sirt1* axis significantly delayed the propagation of *N. caninum* in caprine EECs by regulating host cell mitochondrial function and autophagy.

LncRNAs are usually transcribed by RNA Pol II and undergo extensive alternative splicing to greatly increase the reservoir of transcriptional diversity (32, 33). Emerging evidence suggests that splice variants of lncRNAs are involved in various pathological processes (34–39). For example, lncRNA *SOX2OT* is significantly upregulated in esophageal squamous cell carcinoma and has two splice variants (namely SOX2OT-S1 and SOX2OT-S2). Each variant is markedly increased in sub-G1 phase of cell cycle and is significantly downregulated in the induction of neural differentiation (34). In tongue squamous carcinoma, a hypoxia-associated lncRNA, *LINC00887*, generates two variants (namely 887S and 887L) that share the same downstream target, Carbonic Anhydrase IX (CA9), a well-known hypoxia-induced gene during tumor progression. Interestingly, 887S and 887L interact under normoxia, while upon hypoxia, 887S and 887L have opposite effects on tumor progression (35). The lncRNA *RP11-369C8.1* produces six splice variants, two of which (TRMP and TRMP-S) have been characterized to promote the growth of cancer cells by modulating cell cycle progression through distinct mechanisms of action (38, 39). In the present study, we found that *XR_001919077.1* expresses six splice variants, but all these variants significantly inhibit intracellular propagation of *N. caninum* tachyzoites in caprine EECs.

The functions of lncRNAs are closely related to their subcellular locations. To some extent, lncRNAs located in the nucleus are mainly involved in histone modifications, chromatin remodeling, and scaffolding of nuclear complexes at the epigenetic and transcription levels, while lncRNAs in the cytoplasm participate in mRNA translation and stability and act as miRNA sponges at post-transcriptional and translational programs (6, 40). In the present study, *XR_001919077.1* was found in both the cytoplasm and nucleus of caprine EECs using the FISH assay. Previous studies showed that the most well-known function of lncRNAs in the cytoplasm is to act as ceRNAs or microRNA decoys to modulate gene expression (41–43). For example, the lncRNA *WAC-AS1* is significantly upregulated in serum samples of patients and HepG2 2.15 cells infected with hepatitis B virus (HBV), and this lncRNA induces host cell autophagy to facilitate HBV replication in HepG2.2.15 cells by targeting the miR-192-5p/ATG7 axis (41). LncRNA *DANCR* is significantly upregulated in peripheral blood samples of pulmonary tuberculosis patients and THP-1 cells infected with *Mycobacterium tuberculosis* H37Ra, but it inhibits intracellular survival of *M. tuberculosis* by sponging miR-1301-3p and miR-5194 to induce host cell autophagy (42). In the present study, we find that the expression of *XR_001919077.1* negatively affects the propagation of *N. caninum* tachyzoites by regulating the Chi-miR-93-5p/*sirt1* axis, suggesting the defense role of the *XR_001919077.1*/Chi-miR-93-5p/*sirt1* axis during *N. caninum* infection.

SIRT1 is an important conserved mammalian NAD (+)-dependent protein deacetylase in mitochondrial biogenesis and autophagy (44, 45). The growth of *Leishmania infantum* is significantly reduced in bone marrow macrophages knocked out of SIRT1, which inhibits the metabolic switch from an early aerobic glycolytic environment toward mitochondrial oxidation (44). During infection of *T. gondii*, 4-hydroxybenzaldehyde, an aromatic compound, induces SIRT1-mediating autophagy by increasing the intracellular co-localization of autophagic vacuoles and *T. gondii*-containing parasitophorous vacuoles in bone marrow-derived macrophages, and the SIRT1-mediating autophagy restricts the intracellular propagation of *T. gondii* (45). Our previous study found that downregulation of *sirt1* is beneficial for the propagation of *N. caninum* in caprine EECs by regulating mitochondrial function and autophagy (20). In the present study, we found that *N. caninum* infection induces mitochondrial dysfunction and autophagy to facilitate intracellular propagation through targeting the *XR_001919077.1*/Chi-miR-93-5p/*sirt1* axis.

### Conclusions

**Fig 10:**
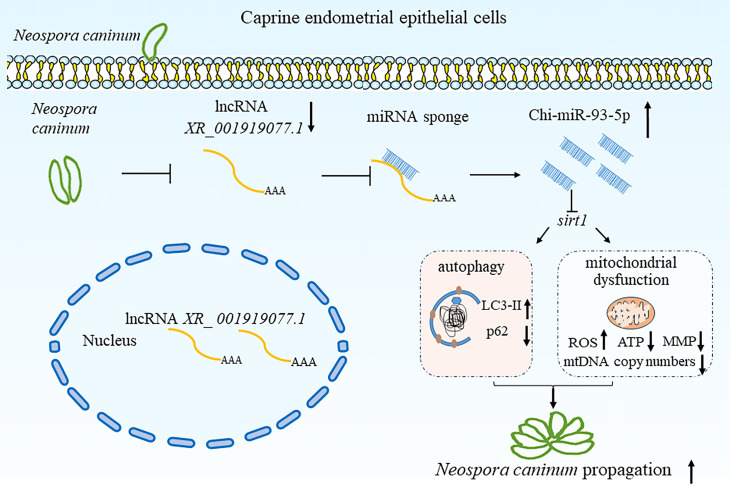
A hypothetical mechanism graph of *XR_001919077.1*/Chi-miR-93-5p/*sirt1* axis regulates the propagation of *N. caninum in vitro. N. caninum* infection significantly downregulates the expression of *XR_001919077.1* and *sirt1* but upregulates the expression of Chi-miR-93-5p. *XR_001919077.1* promotes the expression of *sirt1* by sponging Chi-miR-93-5p in caprine EECs infected with *N. caninum*. The *XR_001919077.1*/Chi-miR-93-5p/*sirt1* axis negatively affects the propagation of *N. caninum* tachyzoites *in vitro* by regulating mitochondrial function and autophagy.

We characterized one caprine lncRNA, *XR_001919077.1*, which is specifically suppressed by *N. caninum* infection, impairing host defense to facilitate parasite propagation (immune evasion by this parasite), and clarified the function of *XR_001919077.1* during *N. caninum* infection. Six splice variants of *XR_001919077.1* are detected, and these variants promote the expression of *sirt1* by sponging Chi-miR-93-5p. The *XR_001919077.1*/Chi-miR-93-5p/*sirt1* axis negatively affects the propagation of *N. caninum* tachyzoites *in vitro* by regulating mitochondrial function and autophagy (Fig. 10). To the best of our knowledge, this is the first study on the significance of lncRNAs during *N. caninum* infection. The findings of this study provide a novel insight to unveil the mechanisms of intracellular survival and pathogenesis of *N. caninum* in reproductive organs/tissues.

## SUPPLEMENTAL MATERIAL

Figure S1 spectrum.01580-24-s0001.tif The expression of *XR_001919077.1* is not altered in caprine endometrial epithelial cells (EECs) infected with *Toxoplasma gondii* or stimulated with lipopolysaccharide (LPS).

Figure S2 spectrum.01580-24-s0002.tif 3' and 5' end sequences of *XR_001919077.1* are obtained by using rapid amplification of cDNA ends (RACE) assays.

Figure S3 spectrum.01580-24-s0003.tif Sequence alignment of six variants of *XR_001919077.1*.

Figure S4 spectrum.01580-24-s0004.tif Expression of each *XR_001919077.1* variant is analyzed.

Figure S5 spectrum.01580-24-s0005.tif *XR_001919077.1* localizes in both cytoplasm and nucleus in caprine endometrial epithelial cells (EECs) infected with *Neospora caninum* at 48 h post infection (hpi).

Figure S6 spectrum.01580-24-s0006.tif The expression of potential sponged miRNAs by *XR_001919077.1* are analyzed in caprine endometrial epithelial cells (EECs) during *N. caninum* infection.

Figure S7 spectrum.01580-24-s0007.tif Transfection efficiencies of Chi-miR-93-5p mimics and Chi-miR-93-5p inhibitor are determined in infected caprine EECs.

Figure S8 spectrum.01580-24-s0008.tif The *XR_001919077.1*/Chi-miR-93-5p axis affects the mRNA levels of *sirt1* in caprine endometrial epithelial cells (EECs) following *N. caninum* infection.

Figure S9 spectrum.01580-24-s0009.tif Transfection efficiency of overexpression plasmid or small interfering RNAs (siRNAs) of *sirt1* is determined.

Table S1 spectrum.01580-24-s0010.xlsx Nucleotide sequences of primers are used in this study.

ASM does not own the copyrights to Supplemental Material that may be linked to, or accessed through, an article. The authors have granted ASM a non-exclusive, world-wide license to publish the Supplemental Material files. Please contact the corresponding author directly for reuse.
