# Supplementary figures and images for: Neospora caninum infection specifically suppresses the expression of a host lncRNA XR_001919077.1 to facilitate parasite propagation by modulating host cell mitochondrial function and autophagy
Source: Microbiol Spectr. 2024 Dec 23;13(2):e01580-24. doi: 10.1128/spectrum.01580-24 (PMC11792476; doi:10.1128/spectrum.01580-24)

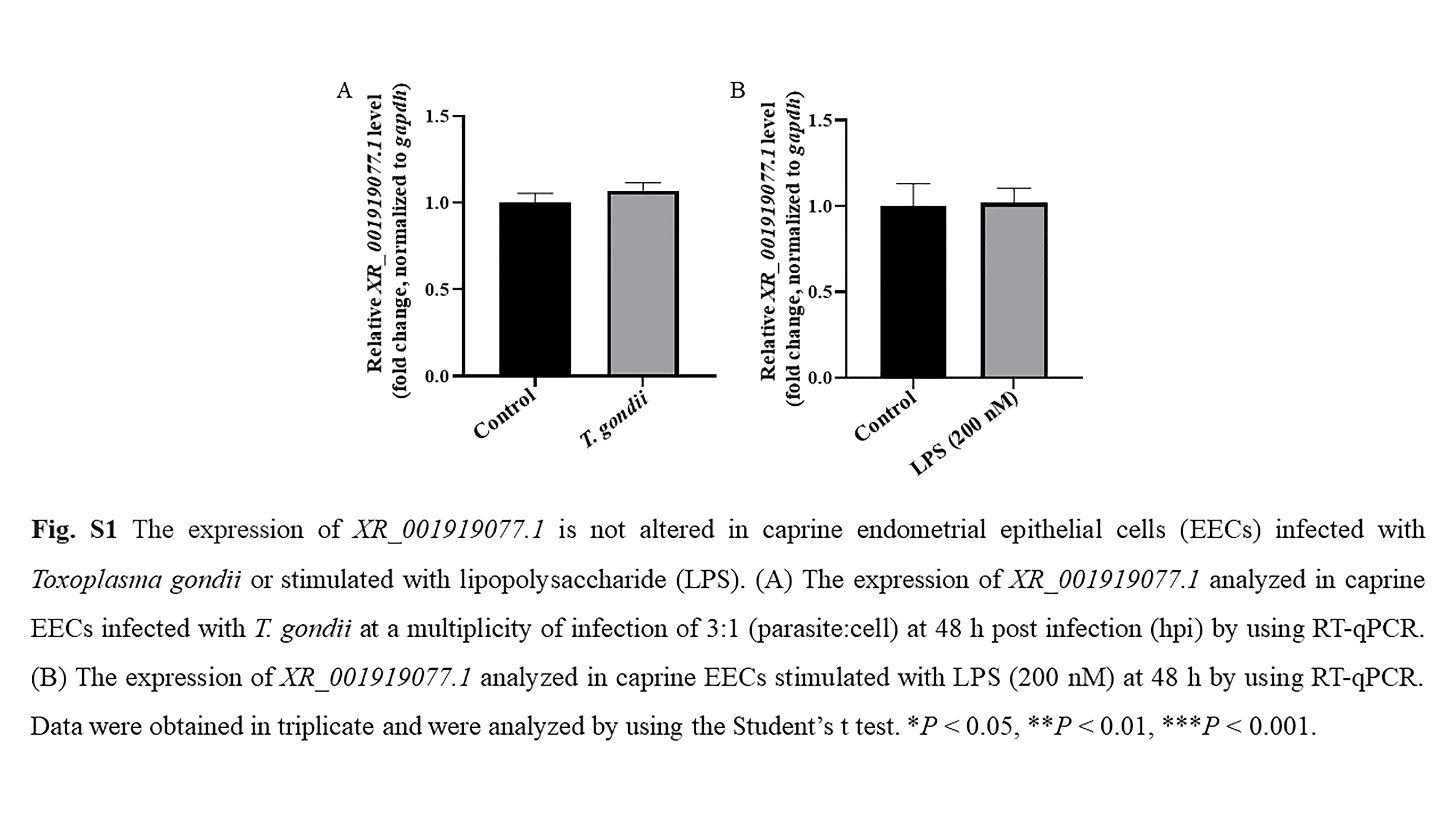

Supplement: Figure S1 — The expression of XR_001919077.1 is not altered in caprine endometrial epithelial cells (EECs) infected with Toxoplasma gondii or stimulated with lipopolysaccharide (LPS). [file spectrum.01580-24-s0001.tif]

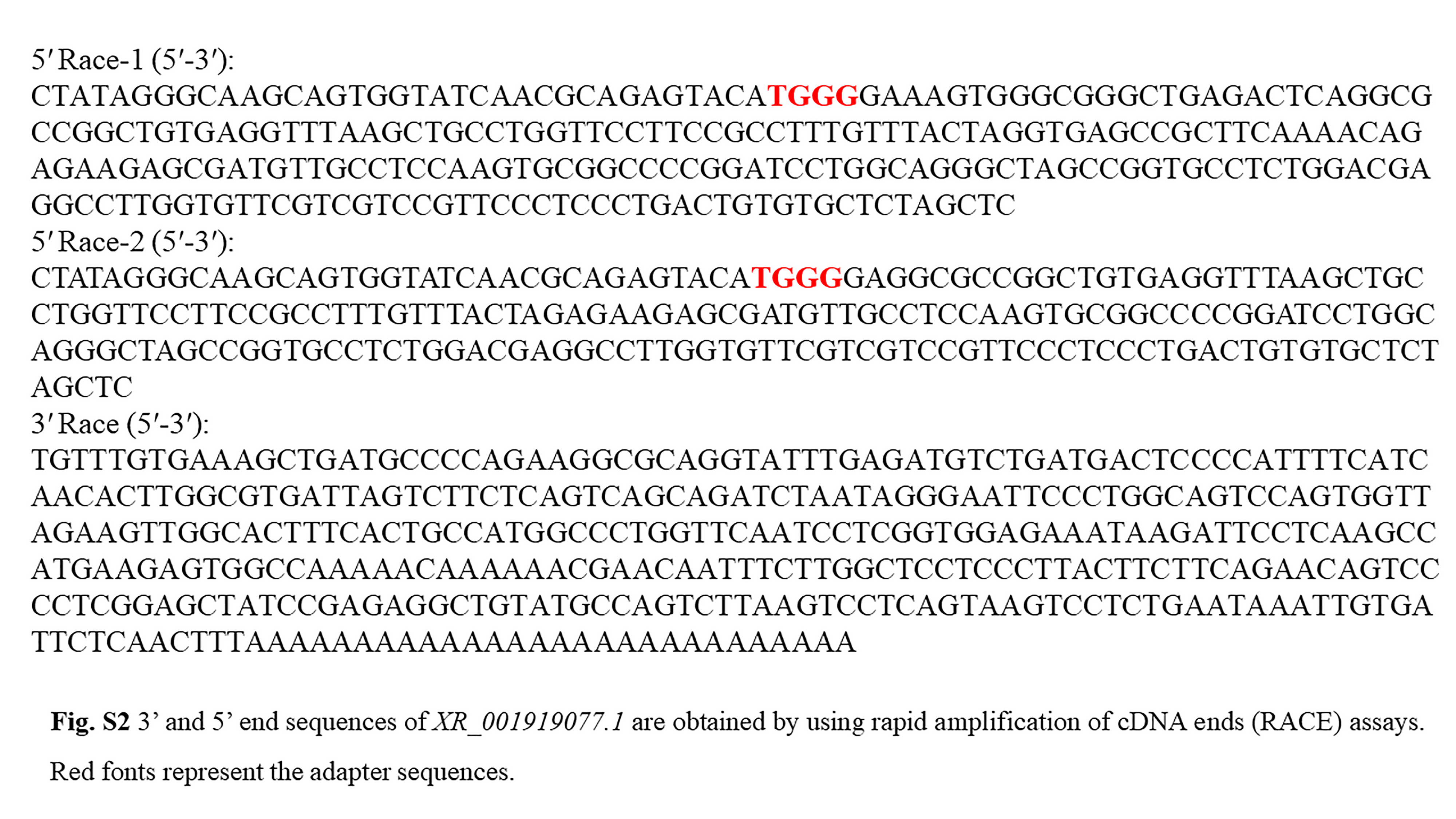

Supplement: Figure S2 — 3' and 5' end sequences of XR_001919077.1 are obtained by using rapid amplification of cDNA ends (RACE) assays. [file spectrum.01580-24-s0002.tif]

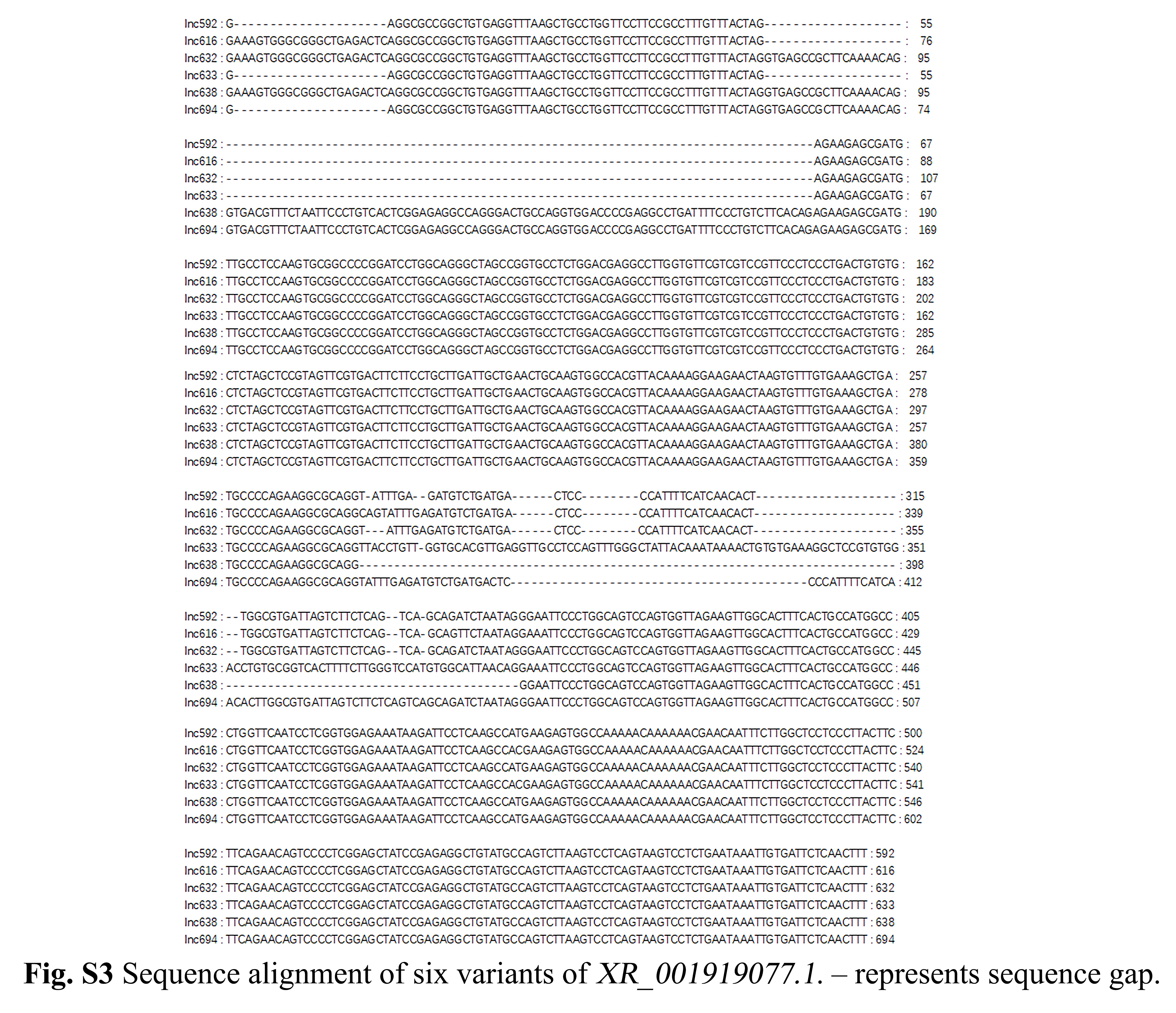

Supplement: Figure S3 — Sequence alignment of six variants of XR_001919077.1. [file spectrum.01580-24-s0003.tif]

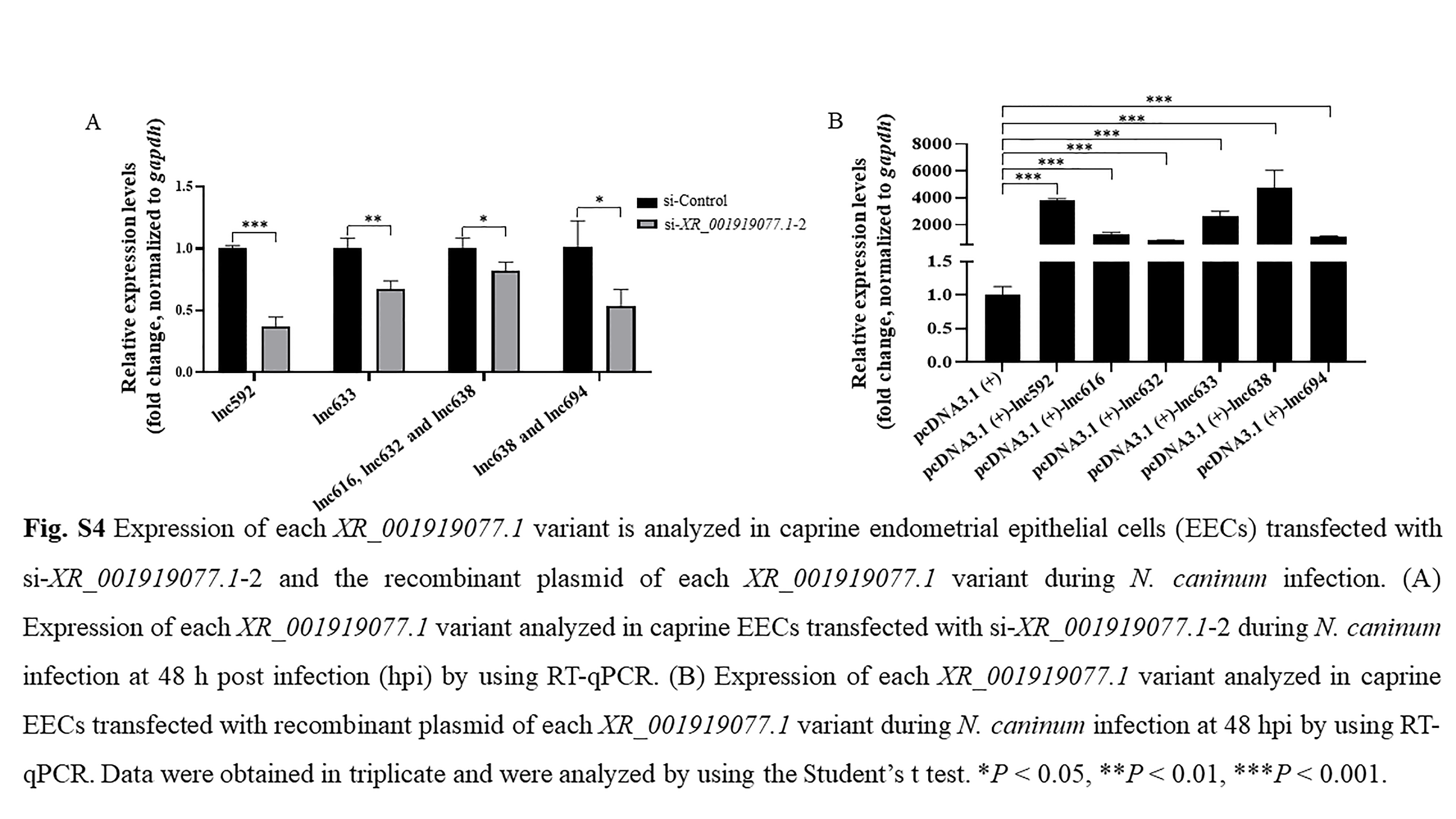

Supplement: Figure S4 — Expression of each XR_001919077.1 variant is analyzed. [file spectrum.01580-24-s0004.tif]

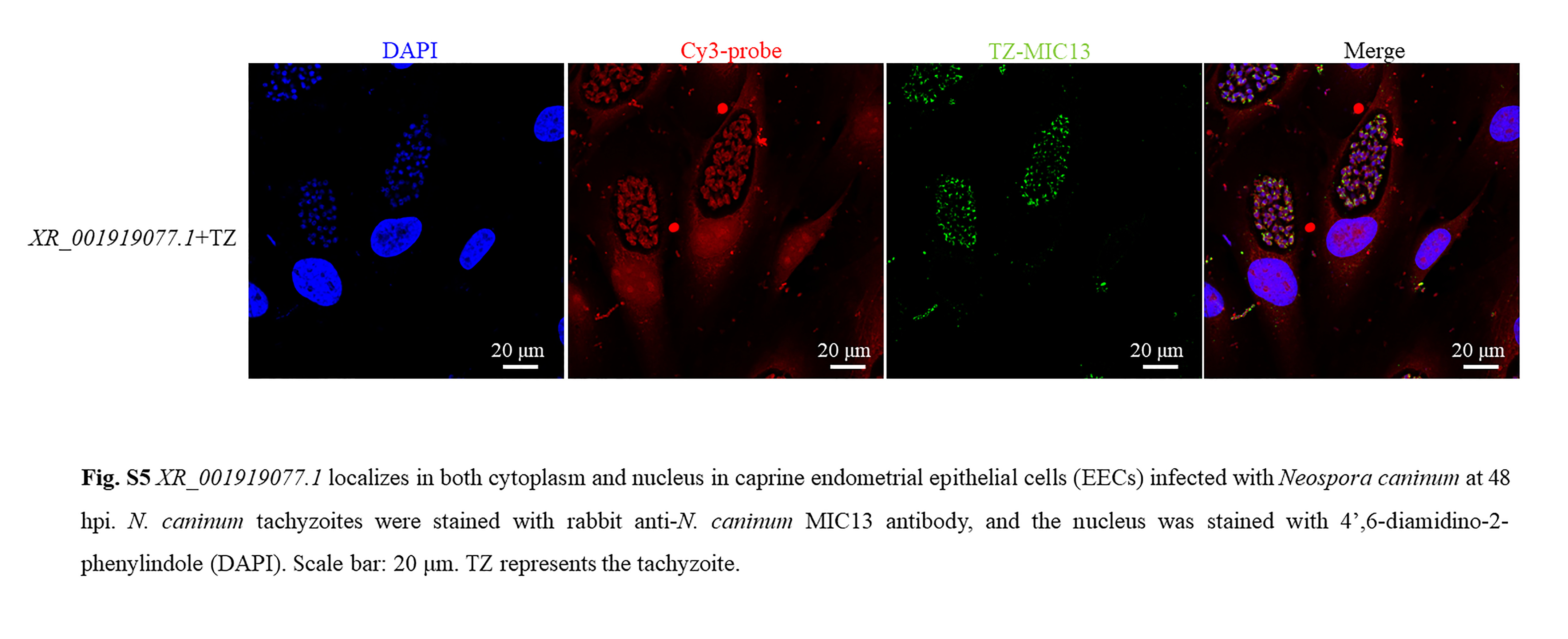

Supplement: Figure S5 — XR_001919077.1 localizes in both cytoplasm and nucleus in caprine endometrial epithelial cells (EECs) infected with Neospora caninum at 48 h post infection (hpi). [file spectrum.01580-24-s0005.tif]

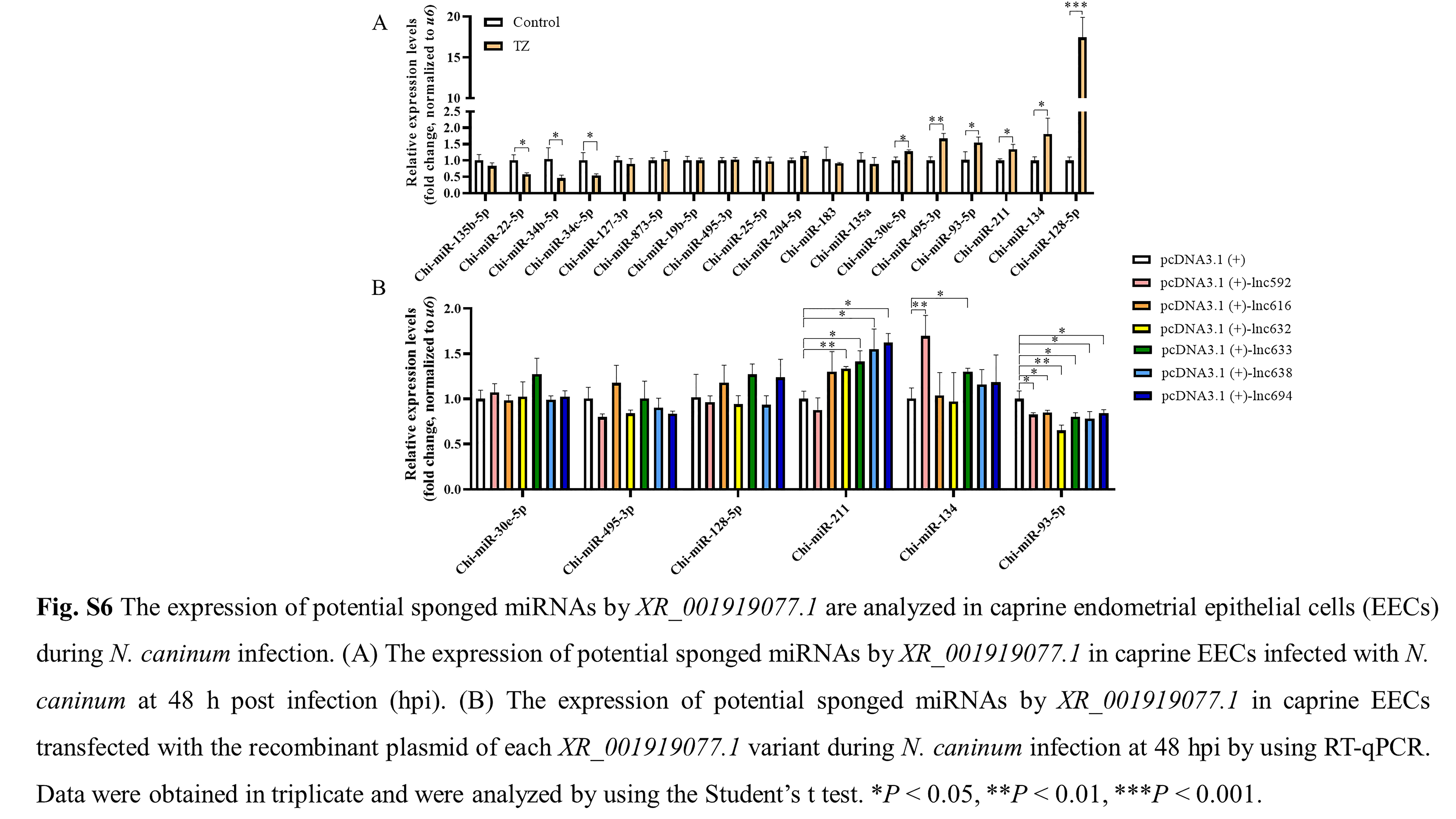

Supplement: Figure S6 — The expression of potential sponged miRNAs by XR_001919077.1 are analyzed in caprine endometrial epithelial cells (EECs) during N. caninum infection. [file spectrum.01580-24-s0006.tif]

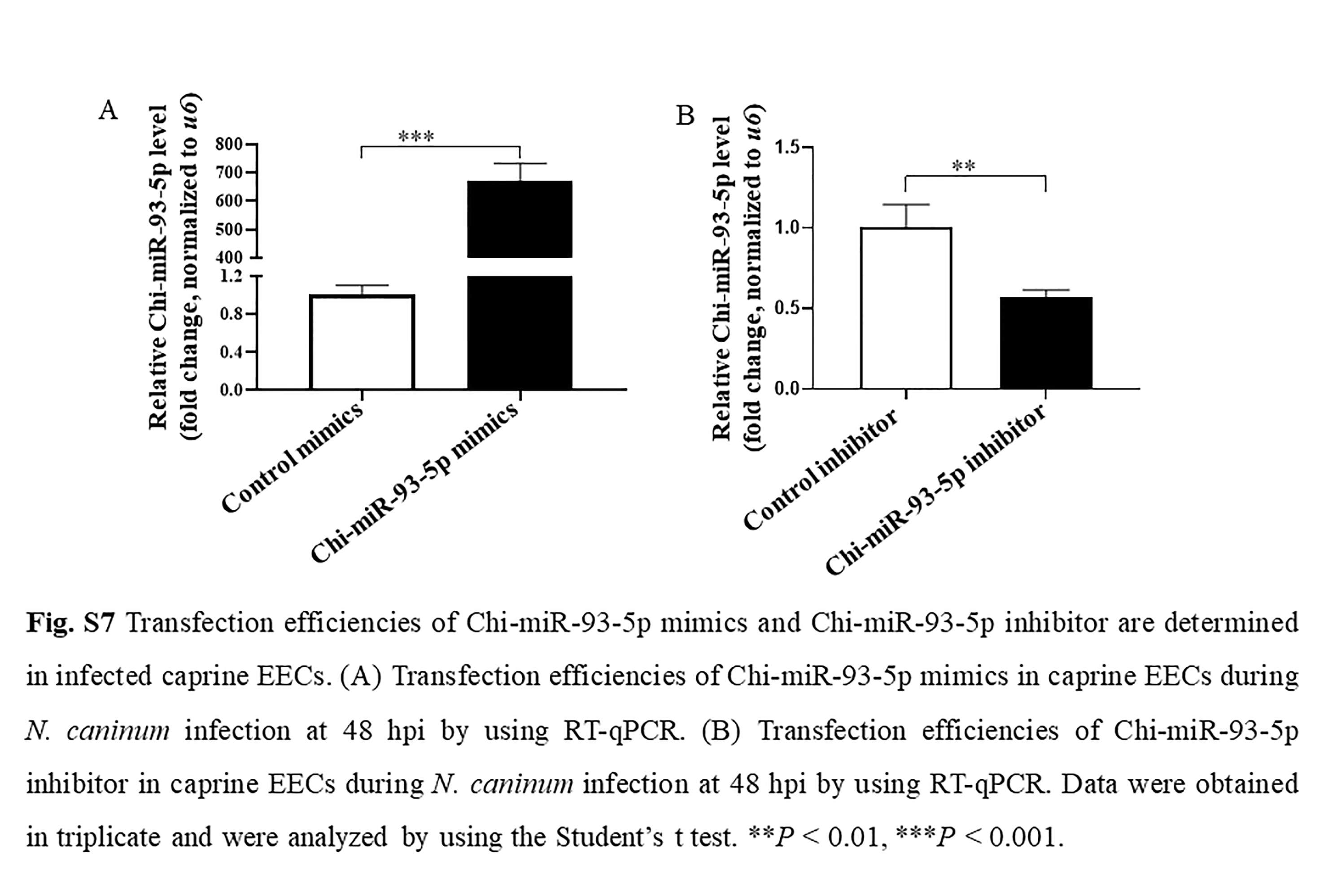

Supplement: Figure S7 — Transfection efficiencies of Chi-miR-93-5p mimics and Chi-miR-93-5p inhibitor are determined in infected caprine EECs. [file spectrum.01580-24-s0007.tif]

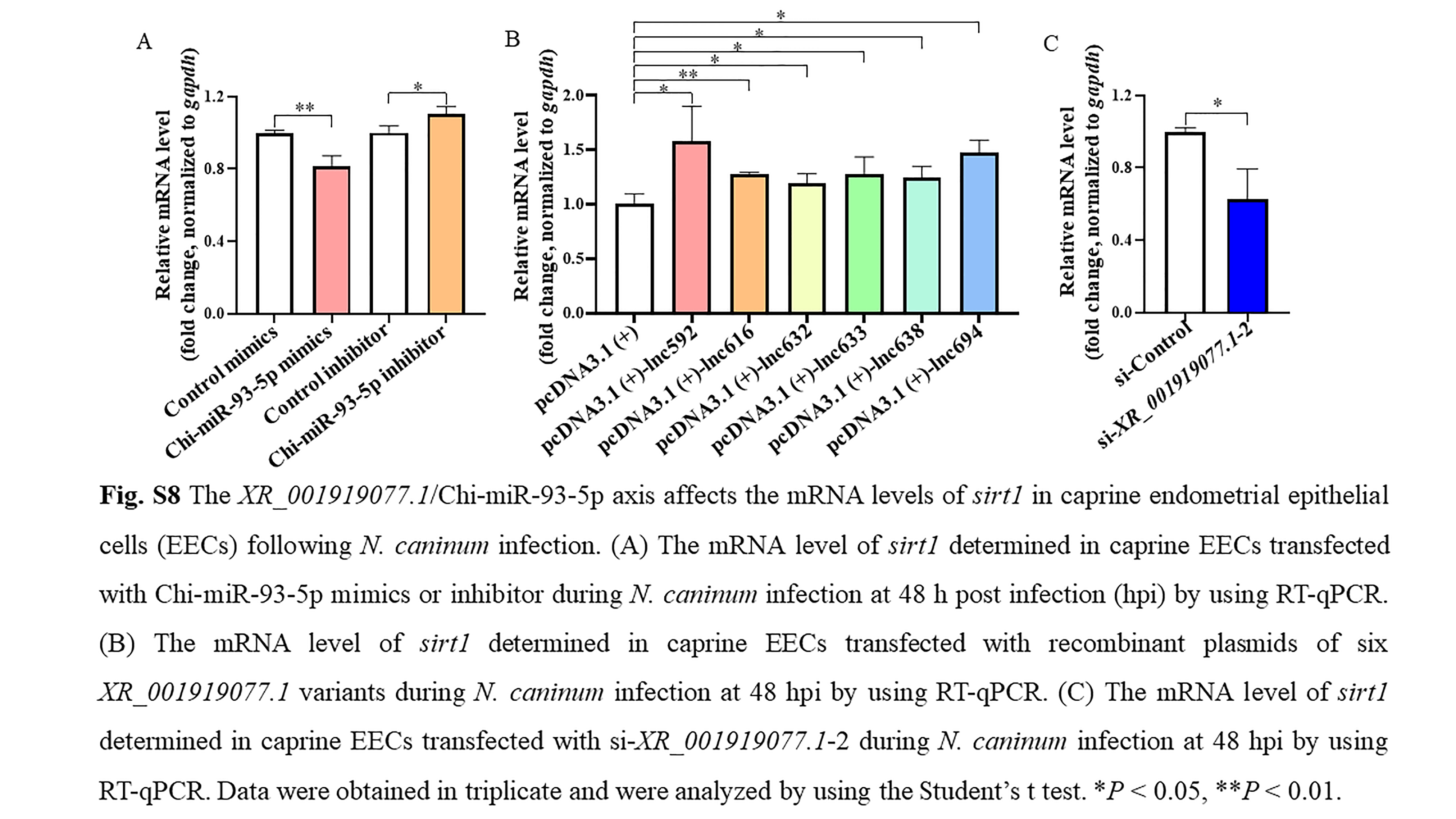

Supplement: Figure S8 — The XR_001919077.1/Chi-miR-93-5p axis affects the mRNA levels of sirt1 in caprine endometrial epithelial cells (EECs) following N. caninum infection. [file spectrum.01580-24-s0008.tif]

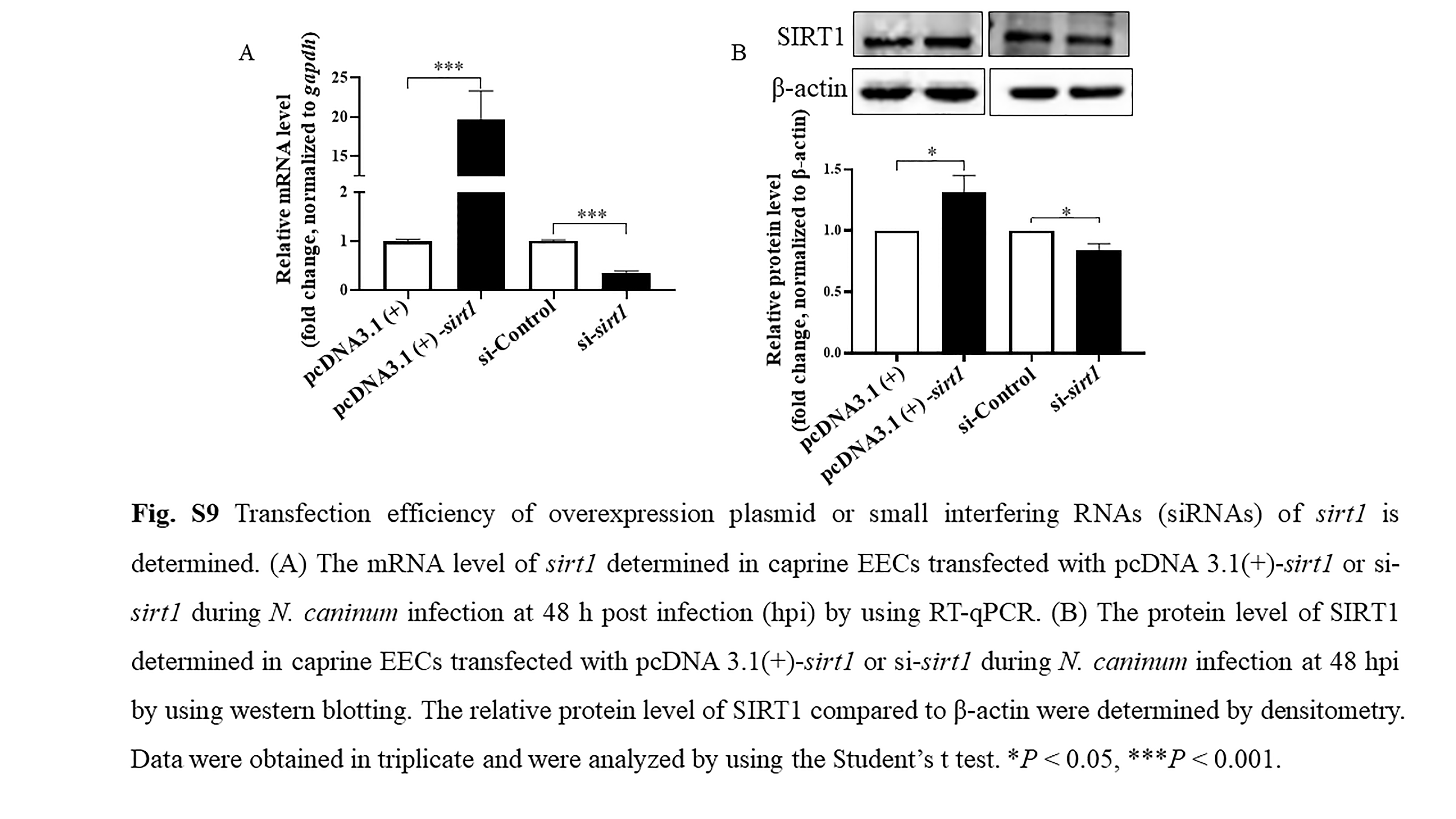

Supplement: Figure S9 — Transfection efficiency of overexpression plasmid or small interfering RNAs (siRNAs) of sirt1 is determined. [file spectrum.01580-24-s0009.tif]
